# Empagliflozin in patients admitted to hospital with COVID-19 (RECOVERY): a randomised, controlled, open-label, platform trial

**DOI:** 10.1016/S2213-8587(23)00253-X

**Published:** 2023-12

**Authors:** RECOVERY Collaborative Group, O Abani, A Abbas, F Abbas, J Abbas, K Abbas, M Abbas, S Abbasi, H Abbass, A Abbott, N Abdallah, A Abdelaziz, M Abdelfattah, B Abdelqader, A Abdul, B Abdul, S Abdul, A Abdul Rasheed, A Abdulakeem, R Abdul-Kadir, A Abdullah, A Abdulmumeen, R Abdul-Raheem, N Abdulshukkoor, K Abdusamad, Y Abed El Khaleq, M Abedalla, A Abeer Ul Amna, L Abel, K Abernethy, M Abeywickrema, C Abhinaya, A Abidin, A Aboaba, A Aboagye-Odei, C Aboah, H Aboelela, H Abo-Leyah, K Abouelela, A Abou-Haggar, M Abouibrahim, A Abousamra, M Abouzaid, M Abraham, T Abraham, A Abraheem, J Abrams, R Abrams, HJ Abu, A Abu-Arafeh, SM Abubacker, A Abung, Y Abusamra, Y Aceampong, A Achara, D Acharya, F Acheampong, P Acheampong, S Acheampong, J Acheson, S Achieng, A Acosta, R Acquah, C Acton, J Adabie-Ankrah, P Adair, AS Adam, F Adam, M Adam, H Adamali, M Adamczyk, C Adams, D Adams, K Adams, L Adams, N Adams, R Adams, T Adams, L Adamu-Ikeme, K Adatia, K Adcock, L Addai-Boampong, A Addo, O Adeagbo, A Adebiyi, O Adedeji, Y Adegeye, K Adegoke, V Adell, S Adenwalla, FW Adeoye, OA Adesemoye, EO Adewunmi, A Adeyanju, J Adeyemi, T Adeyemo, B Adhikari, SA Adhikari, R Adhikary, A Aditya, P Adjepong, G Adkins, A Adnan, M Adriaanse, J Aeron-Thomas, D Affleck, C Afnan, M Afridi, P Afrim, FA Afriyie, ZA Aftab, A Afum-Adjei Awuah, M Agarwal, PN Agasiya, R Agbeko, C Agbo, S Aggarwal, A Aghababaie, L Aguilar Jimenez, JA Agyekum, K Agyen, EK Ahadome, S Ahamed Sadiq, MH Ahammed Nazeer, M Ahmad, S Ahmad, A Ahmed, BAR Ahmed, B Ahmed, F Ahmed, H Ahmed, I Ahmed, K Ahmed, L Ahmed, M Ahmed, MC Ahmed, MS Ahmed, N Ahmed, N Ahmed, O Ahmed, RA Ahmed, R Ahmed, R Ahmed, S Ahmed, SG Ahmed, SH Ahmed, R Ahmed Ali, B Ahmed Mohamud, S Ahmed, S Ahmer, A Ahonia, C Aidoo, C Aiken, D Ail, M Ainsworth, M Aissa, L Aitken, B Ajay, A Ajibode, A Ajmi, N Akhtar, S Akili, B Akinbiyi, O Akindolie, Y Akinfenwa, O Akinkugbe, I Akinpelu, M Akram, O Aktinade, U Akudi, ASAR Al Aaraj, A Al Balushi, M Al Dakhola, A Al Swaifi, E Al-Abadi, A Alabi, N Aladangady, M Alafifi, A Alam, S Alam, A Al-Asadi, K Alatzoglou, P Albert, A Albertus, L Albon, A Alcala, G Alcorn, S Alcorn, A Aldana, D Alderdice, A Aldesouki, R Aldouri, J Aldridge, N Aldridge, RM Ale, A Alegria, A Alexander, C Alexander, J Alexander, PDG Alexander, J Al-fori, L Alghazawi, O Alhabsha, B Al-Hakim, R Alhameed, M Al-Hayali, S Al-Hity, A Ali, FR Ali, J Ali, M Ali, MS Ali, N Ali, O Ali, R Ali, S Ali, E Aliberti, J Alin, A Alina, A Alipustain, B Alisjahbana, F Aliyuda, K Alizadeh, M Al-Jibury, S Al-Juboori, M Al-Khalil, A Alkhudhayri, M Alkhusheh, F Allan, N Allan, A Allanson, R Allcock, E Allen, J Allen, K Allen, L Allen, P Allen, R Allen, S Allen, T Allen, A Alli, K Allison, B Allman, HK Allsop, L Allsop, D Allsup, AFT Almahroos, H Al-Moasseb, M Al-Obaidi, L Alomari, A Al-Rabahi, B Al-Ramadhani, Z Al-Saadi, R Al-Sammarraie, I Alshaer, R Al-Shahi Salman, W Al-Shamkhani, F Alsheikh, B Al-Sheklly, S Altaf, A Alty, M Alvarez, M Alvarez Corral, E Alveyn, M Alzetani, S Amamou, N Amar, S Ambalavanan, R Ambrogetti, C Ambrose, A Ameen, A Amelia Ganefianty, K Ames, MR Amezaga, A Amin, A Amin, K Amin, S Amin, T Amin, B Amit, A Amjad, N Amjad, J Amoah-Dankwa, A Amoako-Adusei, V Amosun, M Amsal, K Amsha, J Amuasi, N Amutio Martin, P Amy, A Anada, A Anand, S Anandappa, SD Anantapatnaikuni, NKN Andari, E Anderson, J Anderson, L Anderson, M Anderson, N Anderson, R Anderson, S Anderson, W Anderson, P Andreou, A Andrews, J Andrews, K Aneke, A Ang, WW Ang, T Angel, A Angela, P Angelini, L Anguvaa, O Anichtchik, M Anim-Somuah, K Aniruddhan, J Annett, L Anning, M Ansah, PJ Anstey, R Anstey, A Anthony, A Anthony-Pillai, P Antill, Z Antonina, V Anu, M Anwar, S Anwar, E Apetri, A Apostolopoulos, S Appleby, D Appleyard, MF Aquino, B Araba, S Aransiola, M Araujo, A Archer, D Archer, S Archer, D Arcoria, C Ardley, G Arhin-Sam, A-M Arias, O Aribike, R Arimoto, NLPE Arisanti, C Arkley, C Armah, I Armata, J Armistead, A Armitage, C Armstrong, M Armstrong, S Armstrong, W Armstrong, P Armtrong, H Arndt, C Arnison-Newgass, D Arnold, R Arnold, A Arnott, D Arora, K Arora, P Arora, R Arora, A Arter, A Arthur, NM Artini, A Arumaithurai, A Arya, R Arya, D Aryal, D Asandei, GA Asare, A Asghar, M Asghar, A Ashab, C Ashbrook-Raby, H Ashby, J Ashcroft, S Ashcroft, G Asher, Z Ashfak, A Ashfaq, HA Asiamah, A Ashish, D Ashley, S Ashman-Flavell, S Ashok, A-E-A Ashour, MZ Ashraf, S Ashraf, MB Ashraq, D Ashton, S Ashton, A Ashworth, FJ Ashworth, R Ashworth, A Aslam, I Aslam, S Aslam, L Aslett, H Asogan, A Asrar, O Assaf, R Astin-Chamberlain, YE Atabudzi, P Athavale, D Athorne, B Atkins, C Atkins, S Atkins, J Atkinson, V Atkinson, A Atomode, B Atraskiewicz, AA Attia, E Attubato, M Attwood, P Aubrey, Z Auer, A Aujayeb, AT Aung, H Aung, HWW Aung, KK Aung, KT Aung, N Aung, Y Aung, ZM Aung, E Austin, K Austin, A Auwal, M Avari, M Avery, N Aveyard, J Avis, G Aviss, C Avram, P Avram, A Awadelkareem, G Awadzi, M Awaly, A Awan, S Awisi, A Aya, E Ayaz, JM Ayerh, A Ayers, J Azam, A Azeem, M Azharuddin, A Aziz, G Aziz, I Aziz, N Aziz, A Azkoul, A Azman Shah, G Azzopardi, H Azzoug, F Babatunde, M Babi, B Babiker, G Babington, M Babirecki, M Babores, AO Babs-Osibodu, T Bac, S Bacciarelli, R Bachar, M-E Bachour, A Bachti, G Bacon, J Bacon, B Badal, A Badat, M Bader, GR Badhan, S Badhrinarayanan, JP Bae, A Baggaley, A Baggott, G Bagley, D Bagmane, L Bagshaw, K Bahadori, Y Bahurupi, A Bailey, J Bailey, K Bailey, L Bailey, MA Bailey, M Bailey, P Bailey, S Bailey, H Baillie, JK Baillie, J Bain, V Bains, D Baird, E Baird, K Baird, S Baird, T Baird, Y Baird, A Bajandouh, DC Baker, E Baker, J Baker, K Baker, M Baker, R Baker, T-A Baker, V Baker, H Bakere, N Bakerly, M Baker-Moffatt, A Bakhai, N Bakhtiar, P Bakoulas, D Bakthavatsalam, N Balachandran, A Balan, P Balasingam, T Balaskas, M Balasubramaniam, N Balatoni, A Balcombe, A Baldwin, A Baldwin, C Baldwin, D Baldwin, F Baldwin, R Baldwin-Jones, N Bale, J Balfour, M Ball, Ro Ball, K Ballard, I Balluz, C Balmforth, E Balogh, A Baltmr, A Baluwala, G Bambridge, A Bamford, P Bamford, A Bamgboye, E Bancroft, H Bancroft, J Banda, K Bandaru, S Bandi, N Bandla, S Bandyopadhyam, A Banerjee, R Banerjee, P Bang, S Baniya, O Bani-Saad, H Banks, L Banks, P Banks, C Bann, H Bannister, O Bannister, L Banton, T Bao, M Baptist, T Baqai, AM Baral, SC Baral, D Baramova, R Barber, E Barbon, M Barbosa, J Barbour, A Barclay, C Barclay, G Bardsley, S Bareford, S Bari, M Barimbing, A Barker, D Barker, E Barker, H Barker, J Barker, L Barker, O Barker, K Barker-Williams, S Barkha, J Barla, G Barlow, R Barlow, V Barlow, J Barnacle, A Barnard, D Barnes, N Barnes, R Barnes, T Barnes, C Barnetson, A Barnett, A Barnett-Vanes, PG Barning, W Barnsley, A Barr, D Barr, J Barr, C Barr, N Barratt, S Barratt, M Barrera, A Barrett, Fi Barrett, J Barrett, S Barrett, E Barrow, J Bartholomew, MS Barthwal, C Bartlett, G Bartlett, J Bartlett, L Bartlett, S Bartley, S Bartolmeu-Pires, A Barton, G Barton, J Barton, L Barton, R Barton, R Baruah, S Baryschpolec, H Bashir, A Bashyal, B Basker, S Basnet, B Basnyat, A Basoglu, A Basran, J Bassett, G Bassett, C Bassford, B Bassoy, V Bastion, A Bastola, A Basumatary, P Basvi, JA Batac, VR Bataduwaarachchi, T Bate, HJ Bateman, K Bateman, V Bateman, E Bates, H Bates, M Bates, S Bates, S Batham, A Batista, A Batla, D Batra, H Batty, T Batty, A Batty, M Baum, R Baumber, C Bautista, F Bawa, T Bawa, FS Bawani, S Bax, M Baxter, N Baxter, Z Baxter, H Bayes, L-A Bayo, F Bazari, R Bazaz, A Bazli, L Beacham, W Beadles, K Beadon, P Beak, A Beale, K Beard, J Bearpark, A Beasley, S Beattie, K Beaumont, D Beaumont-Jewell, T Beaver, S Beavis, C Beazley, S Beck, V Beckett, R Beckitt, S Beckley, H Beddall, S Beddows, D Beeby, S Beeby, G Beech, M Beecroft, N Beer, Sa Beer, J Beety, G Bega, A Begg, S Begg, S Beghini, A Begum, S Begum, S Begum, T Behan, R Behrouzi, J Beishon, C Beith, J Belcher, H Belfield, K Belfield, A Belgaumkar, D Bell, G Bell, J Bell, L Bell, N Bell, P Bell, S Bell, J Bellamu, M Bellamy, T Bellamy, A Bellini, A Bellis, F Bellis, L Bendall, N Benesh, N Benetti, SA Bengu, L Benham, G Benison-Horner, S Benkenstein, T Benn, A Bennett, C Bennett, D Bennett, G Bennett, K Bennett, K Bennett, L Bennett, MR Bennett, S Bennett, K Bennion, G Benoy, V Benson, A Bentley, J Bentley, I Benton, E Beranova, M Beresford, C Bergin, M Bergstrom, J Bernatoniene, T Berriman, Z Berry, F Best, K Best, A-M Bester, Y Beuvink, E Bevan, S Bevins, T Bewick, A Bexley, S Beyatli, F Beynon, A Bhadi, S Bhagani, S Bhakta, R Bhalla, K Bhandal, K Bhandal, A Bhandari, LN Bhandari, S Bhandari, J Bhanich Supapol, A Bhanot, R Bhanot, S Bhasin, A Bhat, P Bhat, R Bhatnagar, K Bhatt, J Bhayani, D Bhojwani, P Bhuie, MS Bhuiyan, S Bhuiyan, A Bibby, F Bibi, N Bibi, S Bibi, T Bicanic, S Bidgood, J Bigg, S Biggs, A Biju, A Bikov, S Billingham, J Billings, P Binh, A Binns, M BinRofaie, O Bintcliffe, C Birch, J Birch, K Birchall, S Bird, M Birt, C Bishop, K Bishop, L Bishop, K Bisnauthsing, N Biswas, M Bittaye, S Biuk, K Blachford, E Black, H Black, K Black, M Black, P Black, V Black, H Blackgrove, B Blackledge, J Blackler, S Blackley, H Blackman, C Blackstock, C Blair, F Blakemore, H Blamey, A Bland, S Blane, S Blankley, P Blaxill, K Blaylock, J Blazeby, N Blencowe, B Bloom, J Bloomfield, A Bloss, A Blowers, S Blows, H Bloxham, S Blrd, L Blundell, A Blunsum, M Blunt, T Blunt, I Blyth, K Blyth, A Blythe, K Blythe, KA Boahen, M Boampoaa, S Board, E Boatemah, B Bobie, K Bobruk, PN Bodalia, N Bodasing, M Boden, T Bodenham, G Boehmer, M Boffito, K Bohanan, K Bohmova, N Bohnacker, S Bokhandi, M Bokhar, S Bokhari, SO Bokhari, I Bokobza, A Boles, C Bolger, C Bonaconsa, C Bond, H Bond, S Bond, T Bond, A Bone, G Boniface, J Bonney, L Bonney, L Booker, S Boot, M Boothroyd, J Borbone, N Borman, S Bosence, K Bostock, N Botting, F Bottrill, H Bouattia, L Bough, H Boughton, Z Boult, T Boumrah, M Bourke, S Bourke, M Bourne, R Bousfield, L Boustred, A Bowes, P Bowker, T Bowker, H Bowler, L Bowman, S Bowman, R Bowmer, A Bowring, H Bowyer, A Boyd, J Boyd, L Boyd, N Boyer, N Boyle, P Boyle, R Boyle, L Boyles, L Brace, A Bracken, J Bradder, CJ Bradley, P Bradley, J Bradley-Potts, L Bradshaw, Z Bradshaw, C Brady, R Brady, S Brady, P Braga Sardo, D Braganza, M Braithwaite, S Brammer, M Branch, T Brankin-Frisby, J Brannigan, S Brattan, F Bray, N Bray, M Brazil, L Brear, Tr Brear, S Brearey, L Bremner, M Brend, C Bresges, C Bressington, G Bretland, C Brewer, M Bridgett, G Bridgwood, S Brigham, J Bright, C Brightling, T Brigstock, L Brimfield, P Brinksman, E Brinkworth, R Brittain-Long, V Britten, H Britton, L Broad, S Broadhead, R Broadhurst, A Broadley, M Broadway, C Brockelsby, M Brocken, T Brockley, M Brodsky, F Brogan, L Brohan, F Brokke, J Brolly, D Bromley, H Brooke-Ball, V Brooker, M Brookes, D Brooking, A Brooks, D Brooks, J Brooks, K Brooks, N Brooks, P Brooks, R Brooks, S Brooks, M Broom, N Broomhead, C Broughton, N Broughton, M Brouns, A Brown, C Brown, E Brown, H Brown, J Brown, L Brown, N Brown, P Brown, R Brown, S Brown, T Brown, B Browne, C Browne, D Browne, M Browne, S Brownlee, A Brraka, J Bruce, M Bruce, W Brudlo, A Brunchi, N Brunskill, A Brunton, M Brunton, M Bryant, E Bryden, H Brzezicki, A Buazon, MH Buch, R Buchan, R Buchanan, D Buche, A Buck, L Buck, M Buckland, C Buckley, L Buckley, P Buckley, S Buckley, C Buckman, A Budds, G Bugg, R Bujazia, M Bukhari, S Bukhari, R Bulbulia, A Bull, D Bull, K Bull, R Bull, Th Bull, E Bullock, S Bullock, N Bulteel, K Bumunarachchi, R Bungue-Tuble, O Burbidge, C Burchett, D Burda, C Burden, TG Burden, Mi Burgess, R Burgess, S Burgess, E Burhan, H Burhan, H Burke, K Burke, A Burman, S Burnard, C Burnett, S Burnett, A Burns, C Burns, J Burns, K Burns, D Burrage, K Burrows, C Burston, A Burton, B Burton, F Burton, H Burton, M Burton, M Butar butar, D Butcher, A Butler, E Butler, J Butler, P Butler, S Butler, J Butler, A-T Butt, M Butt, MM Butt, C Butterworth, N Butterworth-Cowin, R Buttery, T Buttle, H Button, D Buttress, H Bye, J Byrne, W Byrne, V Byrne-Watts, A Cabandugama, L Cabrero, S Caddy, R Cade, A Cadwgan, Z Cahilog, A Cahyareny, D Cairney, J Calderwood, D Caldow, E Cale, G Calisti, D Callaghan, J Callaghan, C Callens, D Callum, C Calver, M Cambell-Kelly, T Camburn, DR Cameron, E Cameron, F Cameron, S Cameron, C Camm, FD Cammack, A Campbell, B Campbell, D Campbell, H Campbell, J Campbell, K Campbell, M Campbell, R Campbell, W Campbell, Q Campbell Hewson, J Camsooksai, L Canclini, SM Candido, J Candlish, C Caneja, A Cann, J Cann, R Cannan, A Cannon, E Cannon, M Cannon, P Cannon, V Cannons, E Canonizado, J Cantliff, N Cap, B Caplin, S Capocci, N Caponi, A Capp, R Capstick, T Capstick, C Caraenache, A Card, M Cardwell, C Carey, R Carey, S Carley, F Carlin, T Carlin, S Carmichael, M Carmody, M Carnahan, C Caroline, J Carpenter, S Carr, A Carrasco, Z Carrington, A Carroll, P Carroll, R Carson, C Cart, E Carter, J Carter, M Carter, N Carter, P Carter, D Cartwright, J-A Cartwright, C Carty, L Carty, J Carungcong, C Carver, E Carver, R Carver, S Casey, A Cassells, T Castiello, G Castle, B Castles, M Caswell, AM Catana, H Cate, A Catelan Zborowski, S Cathcart, K Cathie, D Catibog, C Catley, L Catlow, M Caudwell, A Cavazza, A Cave, L Cave, S Cavinato, F Cawa, K Cawley, C Caws, K Cawthorne, H Cendl, H Century, J Cernova, M Cesay, E Cetti, S Chabane, M Chablani, C Chabo, J Chacko, D Chadwick, J Chadwick, R Chadwick, E Chakkarapani, A Chakraborty, M Chakraborty, M Chakravorty, P Chalakova, BS Chalise, J Chalmers, R Chalmers, G Chamberlain, S Chamberlain, E Chambers, J Chambers, L Chambers, N Chambers, A Chan, C Chan, E Chan, M Chan, K Chan, P Chan, R (P-C) Chan, XHS Chan, C Chandler, H Chandler, KJ Chandler, S Chandler, Z Chandler, S Chandra, N Chandran, B Chandrasekaran, Y Chang, HQ Chanh, G Chaplin, J Chaplin, G Chapman, J Chapman, K Chapman, L Chapman, M Chapman, P Chapman, T Chapman, L Chappell, A Charalambou, B Charles, D Charlton, S Charlton, K Chatar, C Chatha, D Chatterton, N Chau, R Chaube, MYN Chaudhary, B Chaudhary, I Chaudhry, Z Chaudhry, K Chaudhuri, N Chaudhuri, M Chaudhury, A Chauhan, RS Chauhan, A Chavasse, N Chavasse, V Chawla, L Cheater, J Cheaveau, C Cheeld, M Cheeseman, F Chen, HM Chen, T Chen, F Cheng, LY Cheng, Z Cheng, H Chenoweth, CH Cheong, JJ Cherian, S Cherian, M Cherrie, H Cheshire, CK Cheung, E Cheung, K Cheung, M Cheung, C Cheyne, S Chhabra, WL Chia, E Chiang, A Chiapparino, R Chicano, G Chikara, M Chikungwa, ZA Chikwanha, G Chilcott, S Chilcott, A Chilvers, P Chimbo, KW Chin, WJ Chin, R Chineka, A Chingale, E Chinonso, C Chin-Saad, M Chirgwin, H Chisem, C Chisenga, C Chisholm, B Chisnall, C Chiswick, S Chita, N Chitalia, M Chiu, L Chiverton, B Chivima, C Chmiel, S Choi, W Choon Kon Yune, M Chopra, V Choudhary, O Choudhury, S Choudhury, B-L Chow, M Chowdhury, S Chowdhury, A Chrisopoulou, V Christenssen, P Christian, A Christides, F Christie, D Christmas, G Christoforou, T Christopherson, A Christou, M Christy, P Chrysostomou, Y Chua, D Chudgar, R Chudleigh, S Chukkambotla, ME Chukwu, I Chukwulobelu, CY Chung, E Church, SR Church, D Churchill, N Cianci, P Cicconi, P Cinardo, Z Cipinova, B Cipriano, S Clamp, B Clancy, M Clapham, E Clare, S Clare, A Clark, C Clark, D Clark, E Clark, F Clark, G Clark, J Clark, K Clark, L Clark, M Clark, N Clark, P Clark, R Clark, T Clark, Z Clark, A Clarke, J Clarke, P Clarke, R Clarke, S Clarke, A Claxton, L Claxton, K Clay, C Clayton, E Clayton, O Clayton, J Clayton-Smith, B Clearyb, C Cleaver, R Cleeton, I Clement, C Clemente de la Torre, J Clements, S Clements, S Clenton, S Cliff, R Clifford, S Clifford, A Clive, J Clouston, V Clubb, S Clueit, L Clutterbuck, A Clyne, M Coakley, PGL Coakley, K Cobain, A Cochrane, P Cochrane, L Cockayne, M Cockerell, H Cockerill, S Cocks, R Codling, A Coe, S Coetzee, D Coey, D Cohen, J Cohen, O Cohen, M Cohn, L Coke, O Coker, N Colbeck, R Colbert, E Cole, J Cole, G Coleman, M Coleman, N Coleman, H Coles, M Colin, A Colino-Acevedo, J Colley, K Collie, A Collier, D Collier, H Collier, T Collingwood, P Collini, E Collins, J Collins, K Collins, M Collins, N Collins, S Collins, V Collins, A Collinson, B Collinson, J Collinson, M Collis, M Colmar, HE Colton, J Colton, K Colville, C Colvin, E Combes, D Comer, A Comerford, D Concannon, A Condliffe, R Condliffe, E Connell, L Connell, N Connell, K Connelly, G Connolly, E Connor, A Conroy, K Conroy, V Conteh, R Convery, F Conway, G Conway, R Conway, J-A Conyngham, A Cook, C Cook, E Cook, G Cook, H Cook, J Cook, M Cook, S Cook, D Cooke, G Cooke, H Cooke, J Cooke, K Cooke, T Cooke, V Cooke, A Cooper, C Cooper, D Cooper, H Cooper, J Cooper, L Cooper, N Cooper, R Cooper, S Cooray, T Cope, S Corbet, C Corbett, A Corbishley, J Corcoran, C Cordell, J Cordle, A Corfield, J Corless, A Corlett, J Cornwell, M Cornwell, D Corogeanu, A Corr, M Corredera, R Corrigan, P Corry, R Corser, J Cort, D Cosgrove, T Cosier, P Costa, T Costa, C Coston, S Cotgrove, Z Coton, L-J Cottam, R Cotter, D Cotterill, C Cotton, G Couch, M Coulding, A Coull, D Counsell, D Counter, C Coupland, E Courtney, J Courtney, R Cousins, AJ Coutts, A Cowan, E Cowan, R Cowan, R Cowell, L Cowen, S Cowman, A Cowton, E Cox, G Cox, H Cox, K Cox, M Cox, K Coy, A Cradduck-Bamford, H Craig, J Craig, V Craig, F Craighead, M Cramp, H Cranston, SS Crasta, J Crause, A Crawford, E Crawford, I Crawford, S Crawshaw, B Creagh-Brown, A Creamer, A Creaser-Myers, J Cremona, S Cremona, A Crepet, J Cresswell, M Cribb, C Crichton, D Crilly, L Crisp, N Crisp, D Crocombe, M Croft, J Crooks, H Crosby, E Cross, T Cross, A Crothers, S Crotty, S Crouch, M Crow, A Crowder, K Crowley, T Crowley, R Croysdill, C Cruickshank, I Cruickshank, J Cruise, C Cruz, T Cruz Cervera, D Cryans, G Cui, H Cui, L Cullen, G Cummings-Fosong, V Cunliffe, N Cunningham, J Cupitt, H Curgenven, G Curnow, D Curran, S Curran, C Currie, J Currie, S Currie, J Curtis, K Curtis, M Curtis, O Curtis, T Curtis, R Cuthbertson, J Cuthill, S Cutler, S Cutts, M Czekaj, P Czylok, S D'Souza, J da Rocha, GS Dadzie, M Dafalla, A Dagens, H Daggett, J Daglish, S Dahiya, A Dale, K Dale, M Dale, S Dale, J Dales, U D'Alessandro, H Dalgleish, H Dallow, C D'aloia, D Dalton, M Dalton, Z Daly, M Damani, E Damm, L Dan, A Danga, J Dangerfield, A Daniel, P Daniel, A Daniels, A Dann, KG Danso, S Danso-Bamfo, QT Dao, S Darby, A Darbyshire, J Darbyshire, P Dargan, P Dark, K Darlington, S Darnell, T Darton, G Darylile, A Das, M Das, S Das, M Daschel, J Dasgin, D Datta, A Daunt, V Dave, E Davenport, M Davey, M David, A Davidson, L Davidson, N Davidson, R Davidson, A Davies, B Davies, C Davies, D Davies, E Davies, F Davies, G Davies, H Davies, J Davies, K Davies, L Davies, M Davies, N Davies, O Davies, P Davies, R Davies, S Davies, A Davis, J-A Davis, K Davis, P Davis, A Davis-Cook, A Davison, C Dawe, H Dawe, M Dawkins, A Dawson, D Dawson, E Dawson, J Dawson, L Dawson, M Dawson, S Dawson, T Dawson, I Dawson, A Daxter, A Day, J Day, J D'Costa, P De, D de Fonseka, T de Freitas, P De Los Santos Dominguez, R De Pretto, F De Santana Miranda, E de Sausmarez, S de Silva, T de Silva, J De Sousa, P De Sousa, J de Souza, P De Souza, A De Soyza, N de Vere, J de Vos, B Deacon, S Dealing, A Dean, J Dean, K Dean, S Dean, T Dean, J Deane, J Dear, E Dearden, C Deas, S Debbie, G Debreceni, V Deelchand, M Deeley, J Deery, E Defever, M Del Forno, A Dela Rosa, G De-La-Cedra, A Dell, C Demetriou, D DeMets, J Democratis, J Denham, E Denis, L Denley, C Denmade, A Dent, K Dent, M Dent, E Denton, T Denwood, N Deole, D Depala, M Depante, S Dermody, A Desai, P Desai, S Deshpande, V Deshpande, S Devkota, U Devkota, D Devonport, M Devonport, P Dey, V Dey, R Deylami, K Dhaliwal, P Dhangar, S Dhani, A Dhanoa, M Dhar, A Dhariwal, D Dharmasena, D Dhasmana, E Dhillon, R Dhillon, S Dhillon, M Dhimal, D Dhiru, T Dhorajiwala, P Dias, S Diaz, K Diaz-Pratt, M Dibas, D Dickerson, P Dicks, M Dickson, S Dickson, J Digby, R Digpal, S Dillane, S Diment, P Dimitri, G Dimitriadis, S Din, TH Dinh, TTT Dinh, C Dipheko, A Dipper, S Dipro, L Dirmantaite, L Dismore, L Ditchfield, S Diver, L Diwakar, P Diwan, C Dixon, G Dixon, K Dixon, B Djeugam, S Dlamini, P Dlouhy, A D'Mello, P Dmitri, T Do, L Dobbie, M Dobranszky Oroian, C Dobson, L Dobson, M Docherty, D Dockrell, J Dodd, J Dodds, R Dodds, S Dodds, R Dogra, C Doherty, E Doherty, W Doherty, Y Doi, I Doig, E Doke, D Dolan, M Dolman, R Dolman, L Donald, K Donald, C Donaldson, D Donaldson, G Donaldson, K Donaldson, P Dong, M Donkor, S Donlon, J Donnachie, E Donnelly, R Donnelly, P Donnison, A Donohoe, G Donohoe, B Donohue, E Dooks, R Doonan, R Doorn, G Doran, R Dore, K Dorey, S Dorgan, K Dos Santos, M Dosani, D Dosanjh, P Dospinescu, I Doss, T Doudouliaki, A Dougherty, J Doughty, K Douglas, J Douse, A Dow, L Dowden, M Dower, S Dowling, N Downer, C Downes, R Downes, T Downes, D Downey, R Downey, C Downing, L Downs, S Dowson, C Dragan, C Dragos, M Drain, C Drake, V Drew, O Drewett, A Drexel, C Driscoll, H Drogan, O Drosos, G Drummond, K Drury, K Druryk, R Druyeh, J Dryburgh-Jones, S Drysdale, P Dsouza, A Du Thinh, IK Duah, H Dube, J Dube, S Duberley, P Duckenfield, H Duckles-Leech, N Duff, E Duffield, H Duffy, K Duffy, L Dufour, A Duggan, P Dugh, R Duhoky, J Duignan, J Dulay, S Dummer, A Duncan, C Duncan, F Duncan, G Duncan, H Duncan, R Duncan, S Dundas, D Dung, A Dunleavy, J Dunleavy, A Dunn, C Dunn, D Dunn, L Dunn, P Dunn, C Dunne, K Dunne, F Dunning, A Dunphy, TTH Duong, V Duraiswamy, B Duran, I DuRand, L Durdle, N Duric, A Durie, E Durie, S Durogbola, C Durojaiye, L Durrans, K Durrant, H Durrington, I Duru, H Duvnjak, A Dwarakanath, L Dwarakanath, D Dwomoh Nkrumah, E Dwyer, Z Dyar, C Dyball, K Dyer, H Dymond, T Dymond, ED Dzidzomu, C Eades, L Eadie, R Eadie, L Eagles, B Eapen, N Earl, J Early, M Earwaker, N Easom, C East, A Easthope, F Easton, J Easton, P Easton, R Eatough, O Ebigbola, M Ebon, A Eccles, S Eccles, C Eddings, M Eddleston, M Edgar, K Edgerley, N Edmond, M Edmondson, T Edmunds, A Edwards, C Edwards, J Edwards, K Edwards, M Edwards, S Edwards, C Eades, L Eadie, R Eadie, L Eagles, B Eapen, N Earl, J Early, M Earwaker, N Easom, C East, A Easthope, F Easton, J Easton, P Easton, R Eatough, O Ebigbola, M Ebon, A Eccles, S Eccles, C Eddings, M Eddleston, M Edgar, K Edgerley, N Edmond, M Edmondson, T Edmunds, A Edwards, C Edwards, J Edwards, K Edwards, M Edwards, S Edwards, C Eades, L Eadie, R Eadie, L Eagles, B Eapen, N Earl, J Early, M Earwaker, N Easom, C East, A Easthope, F Easton, J Easton, P Easton, R Eatough, O Ebigbola, M Ebon, A Eccles, S Eccles, C Eddings, M Eddleston, M Edgar, K Edgerley, N Edmond, M Edmondson, T Edmunds, A Edwards, C Edwards, J Edwards, K Edwards, M Edwards, S Edwards, L Elliot, A Elliott, F Elliott, K Elliott, S Elliott, A Ellis, C Ellis, K Ellis, L Ellis, R Ellis, T Ellis, Y Ellis, A Ellwood, R Elmahdi, E Elmahi, H-M Elmasry, M El-Naggar, N Elndari, O Elneima, M Elokl, A Elradi, M Elsaadany, MASA Elsayed, S El-Sayeh, H El-Sbahi, M Elsebaei, T Elsefi, K El-Shakankery, A Elsheikh, H El-Taweel, S Elyoussfi, J Emberey, JR Emberson, J Emberton, A Emery, J Emmanuel, I Emmerson, M Emms, F Emond, M Emonts, N Enachi, D Enenche, A Engden, K English, C Enimpah, E Entwistle, H Enyi, M Erotocritou, P Eskander, H Esmail, F Essa, B Evans, C Evans, D Evans, E Evans, G Evans, I Evans, J Evans, L Evans, M Evans, R Evans, S Evans, T Evans, C Everden, S Everden, L Every, H Evison, L Evison, C Ezenduka, J Faccenda, L Fahel, Y Fahmay, I Fairbairn, S Fairbairn, T Fairbairn, A Fairclough, L Fairlie, M Fairweather, A Fajardo, N Falcone, E Falconer, J Fallon, A Fallow, D Faluyi, V Fancois, A Farah, M Farah, Q Farah, NZ Fard, L Fares, A Farg, A Farmer, K Farmer, T Farmery, S Farnworth, F Farook, H Farooq, S Farooq, F Farquhar, H Farr, A Farrell, B Farrell, F Farrukh, J Farthing, S Farzana, R Fasina, A Fatemi, M Fatemi, S Fathima, N Fatimah, M Faulkner, S Faust, C Favager, A Fawad, J Fawke, S Fawohunre, A Fazal, A Fazleen, S Fearby, C Fearnley, A Feben, F Fedel, D Fedorova, C Fegan, M Felongo, L Felton, T Felton, K Fenlon, A Fenn, R Fennelly, I Fenner, C Fenton, M Fenton, G Ferenando, C Ferguson, J Ferguson, K Ferguson, S Ferguson, V Ferguson, D Fernandes, C Fernandez, E Fernandez, M Fernandez, S Fernandez Lopez, J Fernandez Roman, CJ Fernando, J Fernando, A Feroz, P Ferranti, T Ferrari, E Ferrelly, A Ferrera, E Ferriman, S Ferron, N Fethers, B Field, J Field, R Field, K Fielder, L Fieldhouse, A Fielding, J Fielding, S Fielding, A Fikree, S Filipa, S Filson, S Finan, S Finbow, DJ Finch, J Finch, L Finch, S Finch, N Fineman, J Finlayson, L Finlayson, A Finn, J Finn, D Finnerty, C Finney, D Finucane, S Fiouni, J Fiquet, P Firi, J Fisher, N Fisher, D Fishman, K Fishwick, C Fitton, F Fitzgerald, K Fitzjohn, J Flaherty, M Flanagan, C Flanders, N Flaris, G Fleming, J Fleming, L Fleming, P Fleming, W Flesher, A Fletcher, A Fletcher, J Fletcher, L Fletcher, S Fletcher, F Flett, K Flewitt, S Flockhart, C Flood, I Floodgate, J Flor, V Florence, M Flowerdew, S Floyd, MJ Flynn, R Flynn, C Foden, A Fofana, G Fogarty, P Foley, L Folkes, T Fong, DM Font, A Foo, J Foo, A Foot, HR Foot, J Foot, J Forbes, A Ford, J Ford, I Fordham, J Foreman, M Forester, M Forkan, C Fornolles, A Forrest, E Forsey, M Forsey, T Forshall, E Forster, A Forsyth, J Forton, C Foster, E Foster, J Foster, RA Foster, T Foster, A Foulds, I Foulds, F Fowe, N Fowkes, E Fowler, R Fowler, S Fowler, A Fox, C Fox, H Fox, J Fox, L Fox, L Fox, N Fox, O Fox, S Fox, S-J Foxton, E Fraile, R Frake, A Francioni, O Francis, R Francis, S Francis, T Francis-Bacon, H Frankland, J Franklin, S Franklin, C Fraser, A Fratila, S Frayling, M Fredlund, A Freeman, C Freeman, E Freeman, H Freeman, N Freeman, C Freer, E French, T French, K Freshwater, M Frise, R Fromson, A Frosh, J Frost, V Frost, O Froud, R Frowd, A Fryatt, A Frygier, B Fuller, L Fuller, T Fuller, D Fullerton, C Fung, G Fung, S Funnell, J Furness, A Fyfe, N G, E Gabbitas, C Gabriel, Z Gabriel, H Gachi, S Gaffarena, S Gage, J Gahir, S Gajebasia, K Gajewska-Knapik, B Gajmer, Z Galani, C Gale, H Gale, L Gale, R Gale, S Gali, B Gallagher, J Gallagher, R Gallagher, W Gallagher, F Gallam, J Galliford, C Galloway, E Galloway, J Galloway, A Galvin, V Galvis, G Gamble, L Gamble, B Gammon, CN Gan, MB Ganaie, J Ganapathi, R Ganapathy, K Gandhi, S Gandhi, U Ganesh, T Ganeshanathan, S Ganguly, A Gani, P Ganley, U Garcia, E-J Garden, AD Gardener, E Gardiner, M Gardiner, P Gardiner, S Gardiner, C Gardiner-Hill, J Gardner, L Gardner, M Garfield, A Garg, I Garg, N Garlick, D Garner, J Garner, L Garner, Z Garner, R Garr, KA Garrero, M Gartaula, F Garty, R Gascoyne, H Gashau, A Gatenby, E Gaughan, A Gaurav, M Gavrila, J Gaylard, S Gayle, C Geddie, I Gedge, S Gee, F Geele, K Geerthan, M Gellamucho, K Gelly, L Gelmon, S Gelves-Zapata, G Genato, N Gent, S Gent, N Geoghegan, A George, B George, S George, T George, VP George, S Georges, D Georgiou, P Gerard, L Gerdes, L Germain, H Gerrish, A Getachew, L Gethin, S Gettings, H Ghanayem, B Ghavami Kia, S Ghazal, A Gherman, A Ghosh, D Ghosh, J Ghosh, S Ghosh, T Giang, S Giannopoulou, M Gibani, C Gibb, B Gibbison, K Gibbons, A Gibson, B Gibson, J Gibson, K Gibson, S Gibson, M Gigi, C Gilbert, J Gilbert, K Gilbert, B Giles, J Gilham, M Gill, L Gill, P Gillen, A Gillesen, K Gillespie, E Gillham, A Gillian, D Gilliland, R Gillott, D Gilmour, K Gilmour, L Gilmour, L Ginn, F Ginting, T Giokanini-Royal, A Gipson, B Giri, J Girling, R Gisby, A Gkioni, A Gkoritsa, E Gkrania-Klotsas, A Gladwell, J Glanville, J Glasgow, S Glasgow, J Glass, L Glass, S Glaysher, L Gledhill, E Glenday, A Glennon, J Glossop, J Glover, K Glover, M Glover, J Glover Bengtsson, D Glowski, S Glynn, C Gnanalingam, J Goddard, W Goddard, E Godden, J Godden, S Godlee, E Godson, G Godwin, S Gogoi, A Goh, M Gohel, R Goiriz, S Gokaraju, R Goldacre, A Goldsmith, P Goldsmith, D Gomersall, L Gomez, R Gomez-Marcos, A Gondal, C Gonzalez, J Goodall, V Goodall, B Goodenough, A Goodfellow, L Goodfellow, J Goodlife, C Goodwin, E Goodwin, J Goodwin, P Goodyear, R Gooentilleke, M Goonasekara, S Gooseman, S Gopal, C Gordon, S Gordon, R Gore, H Gorick, C Gorman, S Gormely, M Gorniok, D Gorog, M Gorst, T Gorsuch, J Gosai, R Gosling, S Gosling, G Gosney, V Goss, D Gotham, N Gott, E Goudie, N Gould, S Gould, C Goumalatsou, L Gourbault, A Govind, R Govindan, S Gowans, G Gowda, R Gowda, H Gower, P Goyal, S Goyal, C Graham, J Graham, L Graham, R Graham, S Graham, M Graham-Brown, J Grahamslaw, G Grana, T Grandison, L Grandjean, A Grant, D Grant, K Grant, M Grant, P Grant, R Gravell, J Graves, A Gray, C Gray, G Gray, J Gray, K Gray, N Gray, R Gray, S Gray, A Grayson, F Greaves, P Greaves, A Green, AS Green, C Green, CA Green, D Green, F Green, J Green, M Green, N Green, S Green, D Greene, P Greenfield, A Greenhalgh, D Greenwood, S Greer, J Gregory, K Gregory, T Gregory, J Greig, R Grenfell, T Grenier, J Grenville, J Gresty, S Grevatt, G Grey, S Gribben, A Gribbin, A Gribble, N Grieg, D Grieve, B Griffin, D Griffin, M Griffin, S Griffith, A Griffiths, D Griffiths, I Griffiths, M Griffiths, N Griffiths, O Griffiths, S Griffiths, Y Griffiths, S Grigoriadou, S Grigsby, P Grist, E Grobovaite, D Grogono, C Grondin, R Groome, P Grose, L Grosu, J Grounds, M Grout, H Grover, J Groves, N Grubb, J Grundy, F Guarino, S Gudur, J Guerin, S Guettari, S Gulati, V Gulia, H Gunasekara, P Gunasekera, M Gunawardena, K Gunganah, J Gunn, E Gunter, AK Gupta, A Gupta, R Gupta, T Gupta, V Gupta, A Gupta-Wright, V Guratsky, A Gureviciute, S Gurram, A Gurung, B Gurung, L Gurung, S Gurung, S Gurung Rai, H Guth, N Guthrine, S Gyambrah, P Gyanwali, S Gyawali, N Ha, NX Ha, NT Ha, R Habibi, B Hack, J Hackett, P Hackney, C Hacon, A Haddad, D Hadfield, N Hadfield, S Hadfield, M Hadjiandreou, N Hadjisavvas, A Haestier, N Hafiz, R Hafiz-Ur-Rehman, J Hafsa, S Hagan, JW Hague, R Hague, N Haider, K Haigh, V Haile, J Hailstone, C Haines, S Hainey, M Hair, B Hairsine, J Hajnik, D Hake, L Hakeem, A Haldeos, W Halder, E Hale, J Hale, C Halevy, P Halford, W Halford, A Halim, A Hall, C Hall, E Hall, F Hall, H Hall, J Hall, K Hall, L Hall, J Hallas, K Hallas, C Hallett, J Halliday, A Hallman, H Halls, M Hamdollah-Zadeh, IA Hamed-Adekale, B Hameed, M Hameed, R Hamers, I Hamid, M Hamie, R Hamill, B Hamilton, F Hamilton, G Hamilton, L Hamilton, M Hamilton, N Hamilton, S Hamilton, R Hamlin, E Hamlyn, B Hammans, S Hammersley, K Hammerton, B Hammond, E Hammond, L Hammond, S Hammond, F Hammonds, I Hamoodi, K Hampshire, JA Hampson, J Hampson, L Hampson, L Hamzah, J Han, O Hanci, S Hand, L Handayani, J Handford, S Handrean, NK Handzewniak, S Haney, VTK Hang, D Hanh, S Hanif, E Hanison, J Hannah, A Hannington, M Hannun, A Hanrath, H Hanratty, D Hansen, A Hanson, H Hanson, J Hanson, K Hanson, S Hanson, N Hao, A Haqiqi, M Haque, H Harcourt, L Harden, Z Harding, S Hardman, M Hardwick, G Hardy, J Hardy, Y Hardy, K Haresh, R Harford, B Hargadon, J Hargraves, C Hargreaves, A Harin, M Haris, E Harlock, P Harman, T Harman, M Harmer, MA Haroon, C Harper, H Harper, J Harper, P Harper, R Harper, S Harrhy, K Harrington, S Harrington, Y Harrington-Davies, J Harris, L Harris, M-C Harris, N Harris, S Harris, D Harrison, L Harrison, M Harrison, OA Harrison, R Harrison, S Harrison, T Harrison, W Harrison, E Harrod, C Hart, D Hart, J Hartley, L Hartley, R Hartley, T Hartley, W Hartrey, P Hartridge, S Hartshorn, A Harvey, M Harvey, C Harwood, H Harwood, Z Harzeli, B Haselden, H Hasford, K Hashem, M Hashimm, T Hashimoto, I Hashmi, J Haslam, Z Haslam, G Hasnip, A Hassan, Z Hassan, S Hassasing, J Hassell, P Hassell, A Hastings, B Hastings, J Hastings, S Hathaway-Lees, J Hatton, J Hau, M Havinden-Williams, S Havlik, DB Hawcutt, K Hawes, L Hawes, N Hawes, L Hawker, A Hawkins, J Hawkins, N Hawkins, W Hawkins, D Hawley, E Hawley-Jones, E Haworth, AW Hay, C Hay, A Hayat, J Hayat, M-R Hayathu, A Hayes, J Hayes, K Hayes, M Hayes, F Hayes, P Hayle, C Haylett, A Hayman, M Hayman, M Haynes, R Haynes, R Hayre, C Hays, S Haysom, J Hayward, P Haywood, H Haywood Hasford, T Hazelton, P Hazenberg, Z He, E Headon, C Heal, B Healy, JL Healy, A Hearn, D Heasman, A Heath, D Heath, R Heath, D Heaton, A Heavens, K Hebbron, C Heckman, G Hector, S Heddon, A Hedges, K Hedges, C Heeley, E Heeney, R Heinink, R Heire, I Helgesen, J Hemingway, U Hemmila, B Hemmings, S Hemphill, D Hemsley, A Henderson, E Henderson, J Henderson, S Henderson, J Henry, K Henry, L Henry, M Henry, N Henry, D Henshall, G Herdman, R Herdman-Grant, M Herkes, LE Hermans, F Hernandez, E Heron, L Heron, W Herrington, E Heselden, P Heslop, T Heslop, S Hester, E Hetherington, J Hetherington, C Hettiarachchi, P Hettiarachchi, H Hewer, J Hewertson, A Hewetson, S Hewins, N Hewitson, C Hewitt, D Hewitt, R Hewitt, S Hey, RS Heyderman, M Heydtmann, J Heys, J Heywood, M Hibbert, J Hickey, N Hickey, P Hickey, N Hickman, A Hicks, J Hicks, S Hicks, PTT Hien, T Hien, D Higbee, L Higgins, A Higham, M Highcock, J Highgate, M Hikmat, A Hill, H Hill, J Hill, L Hill, P Hill, U Hill, A Hilldrith, C Hillman-Cooper, J Hilton, Z Hilton, S Hinch, A Hindle, E Hindley, A Hindmarsh, P Hine, K Hinshaw, C Hird, C Hirst, L Hirst, J Hives, HM Hlaing, B Ho, DKK Ho, R Ho, L Hoa, M Hoare, D Hobden, G Hobden, M Hobrok, S Hobson, C Hodge, S Hodge, L Hodgen, G Hodgetts, H Hodgkins, S Hodgkinson, D Hodgson, H Hodgson, L Hodgson, S Hodgson, G Hodkinson, K Hodson, M Hodson, A Hogan, M Hogben, L Hogg, L Hoggett, A Holborow, C Holbrook, R Holbrook, C Holden, J Holden, M Holden, S Holden, T Holder, N Holdhof, H Holdsworth, L Holland, M Holland, N Holland, P Holland, S Holland, ML Hollands, E Holliday, K Holliday, M Holliday, N Holling, L Hollos, N Hollos, L Holloway, S Holloway, M Hollowday, M Hollyer, A Holman, A Holmes, M Holmes, R Holmes, K Holroyd, B Holroyd-Hind, L Holt, S Holt, A Holyome, M Home, R Homewood, K Hong, L Hoole, C Hooper, S Hope, B Hopkins, PW Horby, S Horler, A Hormis, D Hornan, N Hornby, T Horne, Z Horne, R Horner, C Horrobin, L Horsford, M Horsford, V Horsham, A Horsley, E Horsley, S Horton, J Hosea, T Hoskins, MS Hossain, R Hossain, M Hough, S Hough, C Houghton, K Houghton, R Houlihan, K Housely, H Houston, R Hovvels, L How, L Howaniec, J Howard, K Howard, L Howard, M Howard, S Howard, R Howard-Griffin, L Howard-Sandy, S Howe, A Howell, M Howells, L Howie, K Howlett, S Howlett, R Howman, J Hrycaiczuk, H Htet, NZ Htoon, S Htwe, Y Hu, COH Huah, S Huang, K Hubbard, A Huckle, S Huda, A Hudak, L Hudig, H Hudson, S Hudson, O Hudson, A Hufton, C Huggins, A Hughes, C Hughes, E Hughes, G Hughes, H Hughes, L Hughes, M Hughes, R Hughes, S Hughes, V Hughes, W Hughes, L Huhn, C Hui, R Hulbert, D Hull, G Hull, R Hull, A Hulme, P Hulme, W Hulse, G Hulston, R Hum, M Hume, C Humphrey, A Humphries, J Humphries, T Hung, C Hunt, F Hunt, J Hunt, K Hunt, L Hunt, M Hunt, S Hunt, A Hunter, C Hunter, E Hunter, K Hunter, N Hunter, R Hunter, S Hunter, G Huntington, F Huq, E Hurditch, J Hurdman, C Hurley, K Hurley, MA Husain, S Husaini, C Huson, A Hussain, C Hussain, I Hussain, M Hussain, R Hussain, S Hussain, Y Hussain, M Hussam El-Din, SFEM Hussein, Z Hussein, R Hussey, AH Hussien, A Hutchinson, C Hutchinson, D Hutchinson, E Hutchinson, J Hutchinson, C Hutsby, P Hutton, NQ Huy, N Huyen, T Huyen, TB Huyen, NT Huyen Thuong, H Huynh, D Hydes, J Hyde-Wyatt, N Hynes, M Hyslop, A Iakovou, K Ibison, M Ibraheim, A Ibrahim, J Ibrahim, M Ibrahim, W Ibrahim, B Icke, AI Idowu, M Idrees, N Idrees, H Iftikhar, M Iftikhar, C Igwe, O Igwe, M Ijaz, A Ikomi, C Iles, S Iliodromiti, M Ilsley, L Ilves, FM Ilyas, L Imam-Gutierrez, M Iman, C Imray, H Imtiaz, J Ingham, R Ingham, T Ingle, J Inglis, S Inglis-Hawkes, A Ingram, L Ingram, T Ingram, N Innes, P Inns, V Inpadhas, K Inweregbu, AA Ionescu, A Ionita, I Iordanov, A Ipe, J Iqbal, M Iqbal, F Iqbal Sait, I Irabor, J Irisari, R Irons, M Irshad, MS Irshad, J Irvine, V Irvine, R Irving, M Ishak, E Isherwood, G Isitt, A Islam, MDT Islam, S Islam, A Ismail, O Ismail, C Ison, M Israa, S Isralls, H Istiqomah, M Ivan, C Ivenso, N Ivin, A Ivy, S Iwanikiw, K Ixer, M Iyer, A Iakovou, K Ibison, M Ibraheim, A Ibrahim, J Ibrahim, M Ibrahim, W Ibrahim, B Icke, AI Idowu, M Idrees, N Idrees, H Iftikhar, M Iftikhar, C Igwe, O Igwe, M Ijaz, A Ikomi, C Iles, S Iliodromiti, M Ilsley, L Ilves, FM Ilyas, L Imam-Gutierrez, M Iman, C Imray, H Imtiaz, J Ingham, R Ingham, T Ingle, J Inglis, S Inglis-Hawkes, A Ingram, L Ingram, T Ingram, N Innes, P Inns, V Inpadhas, K Inweregbu, AA Ionescu, A Ionita, I Iordanov, A Ipe, J Iqbal, M Iqbal, F Iqbal Sait, I Irabor, J Irisari, R Irons, M Irshad, MS Irshad, J Irvine, V Irvine, R Irving, M Ishak, E Isherwood, G Isitt, A Islam, MDT Islam, S Islam, A Ismail, O Ismail, C Ison, M Israa, S Isralls, H Istiqomah, M Ivan, C Ivenso, N Ivin, A Ivy, S Iwanikiw, K Ixer, M Iyer, K Jabbar, C Jack, J Jackman, S Jackman, A Jackson, B Jackson, E Jackson, H Jackson, L Jackson, M Jackson, N Jackson, S Jackson, Y Jackson, J Jacob, P Jacob, R Jacob, N Jacques, H Jadhav, A Jafar, D Jafferji, A Jaffery, C Jagadish, V Jagannathan, A Jagne, M Jagpal, N Jain, S Jain, S Jaiswal, D Jajbhay, T Jaki, P Jali, B Jallow, Y Jaly, R Jama, A Jamal, S Jamal, Z Jamal, Y Jameel, A James, C James, K James, L James, M James, N James, O James, P James, R James, S James, T James, J Jameson, L Jamieson, A Jamison, P Jane, K Janes, A Janmohamed, D Japp, P Jaques, V Jardim, C Jardine, C Jarman, E Jarnell, E Jarvie, C Jarvis, R Jarvis, P Jastrzebska, H Javed, A Javier, M Jawad, L Jawaheer, A Jayachandran, D Jayachandran, A Jayadev, A Jayakumar, D Jayaram, R Jayaram, G Jayasekera, T Jayatilleke, A Jayebalan, J Jeater, S Jeddi, V Jeebun, MS Jeelani, K Jeffery, H Jeffrey, R Jeffrey, N Jeffreys, B Jeffs, C Jeffs, JP Jeganathan Ponraj, D Jegede, T Jemima, I Jenkin, A Jenkins, C Jenkins, D Jenkins, E Jenkins, I Jenkins, P Jenkins, S Jenkins, F Jennings, J Jennings, L Jennings, V Jennings, E Jerome, D Jerry, G Jervis, E Jessup-Dunton, J Jesus Silva, C Jetha, K Jethwa, RK Jha, S Jhanji, K Jian, Z Jiao, L Jimenez, A Jimenez Gil, J Jith, T Joefield, N Johal, S Johal, K Johannessen, A Johari, A John, M John, N John, E Johns, M Johns, A Johnson, E Johnson, G Johnson, K Johnson, L Johnson, M Johnson, N Johnson, O Johnson, R Johnson, B Johnston, C Johnston, J Johnston, S Johnston, V Johnston, D Johnstone, E Johnstone, J Johnstone, M Joishy, A Jones, B Jones, C Jones, CE Jones, D Jones, E Jones, G Jones, J Jones, K Jones, KE Jones, L Jones, LM Jones, M Jones, N Jones, O Jones, P Jones, PH Jones, R Jones, RE Jones, S Jones, T Jones, R Jonnalagadda, R Jordache, M Jordan, S Jordan, A Jose, L Jose, S Jose, A Joseph, G Joseph, PA Joseph, R Joseph, S Joseph, D Joshi, M Joshi, P Joshi, T Joshi, B Josiah, DK Joy, L Joy, T Joyce, H Ju, A Ju Wen Kwek, A Judd, E Jude, P Judge, J Juhl, S Jujjavarapu, M Juniper, E Juszczak, D Jyothish, K Jabbar, C Jack, J Jackman, S Jackman, A Jackson, B Jackson, E Jackson, H Jackson, L Jackson, M Jackson, N Jackson, S Jackson, Y Jackson, J Jacob, P Jacob, R Jacob, N Jacques, A Jafar, D Jafferji, A Jaffery, C Jagadish, V Jagannathan, A Jagne, M Jagpal, N Jain, S Jain, S Jaiswal, D Jajbhay, T Jaki, B Jallow, Y Jaly, R Jama, A Jamal, S Jamal, Z Jamal, Y Jameel, A James, C James, K James, L James, M James, N James, O James, P James, R James, S James, T James, J Jameson, L Jamieson, A Jamison, P Jane, K Janes, A Janmohamed, D Japp, P Jaques, V Jardim, C Jardine, C Jarman, E Jarnell, E Jarvie, C Jarvis, R Jarvis, P Jastrzebska, H Javed, A Javier, M Jawad, L Jawaheer, A Jayachandran, D Jayachandran, A Jayadev, A Jayakumar, D Jayaram, R Jayaram, G Jayasekera, T Jayatilleke, A Jayebalan, J Jeater, S Jeddi, V Jeebun, MS Jeelani, K Jeffery, H Jeffrey, R Jeffrey, N Jeffreys, B Jeffs, C Jeffs, JP Jeganathan Ponraj, D Jegede, T Jemima, I Jenkin, A Jenkins, C Jenkins, D Jenkins, E Jenkins, I Jenkins, P Jenkins, S Jenkins, F Jennings, J Jennings, L Jennings, V Jennings, E Jerome, D Jerry, G Jervis, E Jessup-Dunton, J Jesus Silva, C Jetha, K Jethwa, RK Jha, S Jhanji, K Jian, Z Jiao, L Jimenez, A Jimenez Gil, J Jith, T Joefield, N Johal, S Johal, K Johannessen, A Johari, A John, M John, N John, E Johns, M Johns, A Johnson, E Johnson, G Johnson, K Johnson, L Johnson, M Johnson, N Johnson, O Johnson, R Johnson, B Johnston, C Johnston, J Johnston, S Johnston, V Johnston, D Johnstone, E Johnstone, J Johnstone, M Joishy, A Jones, B Jones, C Jones, CE Jones, D Jones, E Jones, G Jones, J Jones, K Jones, KE Jones, L Jones, LM Jones, M Jones, N Jones, O Jones, P Jones, PH Jones, R Jones, RE Jones, S Jones, T Jones, R Jonnalagadda, R Jordache, M Jordan, S Jordan, A Jose, L Jose, S Jose, A Joseph, G Joseph, PA Joseph, R Joseph, S Joseph, D Joshi, M Joshi, P Joshi, T Joshi, B Josiah, L Joy, T Joyce, H Ju, A Ju Wen Kwek, A Judd, E Jude, P Judge, J Juhl, S Jujjavarapu, M Juniper, E Juszczak, D Jyothish, K Kabiru Dawa, M Kacar, D Kadad, N Kadam, N Kader, A Kailey, M Kain, G Kakoullis, A Kakrani, A Kala Bhushan, RJK Kalayi, R Kaliannan Periyasami, D Kalita, I Kalla, E Kallistrou, T Kalmus Eliasz, S Kalsoom, E Kam, J Kamara, A Kamath, P Kamath, R Kamath, SA Kamerkar, N Kametas, M Kamfose, C Kamundi, D Kanabar, L Kane, S Kanitkar, O Kankam, T Kannan, A Kant, V Kapil, R Kapoor, S Kapoor, S Kaprapina, S Kar, J Kara, E Karbasi, S Karelia, R Kark, A Karkey, A Karki, S Karmali, V Karunanithi, N Karunaratne, N Kasianczuk, A Kasiappan Balasubramanian, V Kasipandian, R Kassam, J Kathirgamachelvam, M Kati, V Katsande, K Kaul, D Kaur, D Kaur, J Kaur, S Kaur, Z Kausar, L Kavanagh, s Kavanagh, MAA Kawser, A Kay, J Kay, R Kay, S Kay, JN Kayappurathu, S Kayastha, C Kaye, A Kazeem, P KC, M Ke, T Keady, R Kearns, N Kearsley, J Keating, L Keating, E Keddie-Gray, B Keegan, R Keen, N Keenan, J Kefas, S Kegg, L Keith, U Keke, J Kellett, J Kelliher, A Kelly, D Kelly, E Kelly, H Kelly, L Kelly, M Kelly, R Kelly, S Kelly, M Kelly-Baxter, O Kelsall, M Keltos, T Kemp, E Kendall, A Kendall-Smith, S Kennard, A Kennedy, J Kennedy, M Kennedy, S Kennedy-Hay, J Kenny, M Kent, L Keogan, A Keough, D Kernaghan, A Kerr, C Kerrison, A Kerry, H Kerslake, I Kerslake, H Kerss, J Keshet-Price, E Kestelyn, G Keyte, A Khadar, D Khadka, P Khairunnisa, A Khalid, H Khalid, M Khalid, MU Khalid, S Khalid, T Khalifa, A Khalil, S Khalil, A Khan, B Khan, F Khan, M Khan, K Khan, M Khan, MA Khan, N Khan, O Khan, R Khan, S Khan, T Khan, W Khan, Z Khan, MS Khan Tharin, U Khatana, J Khatri, H Khatun, T Khatun, M Kheia, J Khera, D Khiem, HHE Khin, N Khoja, K Khokhar, MQ Khong, J Khoo, C Khurana, J Kibaru, F Kibutu, A Kidd, M Kidd, J Kidney, S Kidney, W Kieffer, T Kien, J Kilbane, C Kilby, E Killen, B Kilner, S Kilroy, B Kim, JW Kim, M Kim, A Kimber, S Kimber, A King, B King, H King, J King, K King, M King, R King, S King, V King, E King-Oakley, L Kingsmore, DJ Kinnear, F Kinney, S Kiran, A Kirby, A Kirk, J Kirk, A Kirkby, E Kirkham, G Kirkman, L Kirkpatrick, U Kirwan, T Kitching, L Kitto, L Kittridge, T Kjoa, S Klaczek, F Kleemann, S Kmachia, CP Knapp, L Knibbs, A Knight, F Knight, M Knight, S Knight, T Knight, E Knights, J Knights, M Knolle, P Knopp, C Knowles, K Knowles, L Knowles, E Knox, L Knox, O Koch, M Kocsor, R Kodituwakku, G Koduri, YJ Koe, J Koirala, A Koirata, E Kolakaluri, M Kolodziej, E Kolokouri, S Kon, N Konar, M Kononen, A Konstantinidis, R Kontogonis, H Koo, I Koopmans, E Kopyj, L Korcierz, J Korolewicz, G Koshy, C Kosmidis, C Kosztolanyi, J Kotecha, E Kothandaraman, R Kothavale, K Koukou, A Kountourgioti, K Kouranloo, R Kousar'c, M Kousteni, A Koutalopoula, M Kovac, A Kozak Eskenazia, K Krasauskas, R Krishnamurthy, V Krishnamurthy, M Krishnan, H Krishnan, N Krishnapalli, S Krizak, S Krueper, S Krupej, J Krzowski, R Kubaisi, S Kubheka, A Kubisz-Pudelko, S Kuckreja, S Kudsk-Iversen, A Kudzinskas, C Kukadiya, N Kulkarni, S Kumala Dewi, M Kuma-Mintah, A Kumar, G Kumar, M Kumar, R Kumar, S Kumar, V Kumar, P Kumar Panda, A Kundu, H Kunst, SS Kunwar, K Kupiec, A Kurani, M Kurdy, K Kuriakose, R Kurian, V Kurmars, C Kuronen-Stewart, RS Kusangaya, V Kushakovsky, A Kutera, A Kuverji, A Kyei-Mensah, H Kyepa, T Kyere-Diabour, M Kyi, NM Kyi, L Kyle, K-T Kyriaki, J Labao, L Labuschagne, L Lacey, N Lack, M Lacson, Z Ladan, E Ladlow, H Lafferty, A Lagnado, S Laha, S Lahane, C Lai, J Lai, P Laidler, R Laing, I Laing-Faiers, E Laity, K Lake, N Lakeman, D Lalloo, F Lalloo, A Lam, C Lam, F Lamb, L Lamb, T Lamb, O Lambert, P Lambert, C Lameirinhas, MKG Lami, H Lamont, M Lamparski, D Lamrani, C Lanaghan, I Lancona-Malcolm, G Landers, MJ Landray, M Lane, N Lane, A Lang, S Lang, D Langer, M Langley, C Langoya, E Langridge, E Langthorne, H Langton, B Lara, T Large, LN Lartey, S Lassa, A Last, S Latham, V Latham, A Latheef, L Latif, N Latt, C Lau, D Lau, E Lau, GG Laura, M Laurenson, E Lavington, H Law, J Law, KY Law, P Law, R Law, L Lawless, C Lawrence, E Lawrence, G Lawrence, HM Lawrence, N Lawrence, R Lawrie, L Lawson, N Lawson, R Lawson, M Lay, S Laybourne, C Laycock, R Layug, M Lazo, TT Le, V Le, A Lea, W Lea, LE Leach, I Leadbitter, T Leahy, R Lean, L Leandro, D Leaning, R Leary, S Leason, MA Ledingham, C Lee, E Lee, H Lee, I Lee, J Lee, S Lee, SH Lee, T Lee, X Lee, R Lee, D Lees, J Lees, H Legge, J Leggett, K Leigh-Ellis, D Leitch, N Leitch, E Lekoudis, P Lemessy, N Lemoine, R Lenagh, K Leng, K Lennon, L Lennon, B Leonard, K Leonard, W Leong, N Leopold, O Lepiarczyk, I Leslie, EN Lestari, E Lester, E Levell, C Levett, A Levynska, A Lewin, A Lewis, C Lewis, D Lewis, H Lewis, J Lewis, K Lewis, L Lewis, M Lewis, N Lewis, R Lewis, C Lewis-Clarke, A Lewszuk, P Lewthwaite, S Ley, A Liao, V Licence, D Lieberman, S Liebeschuetz, T Light, N Lightfoot, P Lillie, A Lillis, B Lim, C Lim, ET Lim, I Lim, T Lim, W Lim, WS Lim, J Limb, D Limbu, U Limbu, C Linares, D Linden, G Lindergard, K Lindley, C Lindsay, E Lindsay, M Lindsay, H Lindsay- Clarke, M Ling, C Lingam, L Linkson, T Linn, M Linney, C Lippold, G Lipscomb, K Lipscomb, L Lipskis, A Lisboa, E Lister, J Little, S Little, L Littlejohn, S Liu, X Liu, DK Llanera, R Llewellyn, M Llewelyn, A Lloyd, O Lloyd, R Lloyd, S Lo, D Loader, C Loan, L Lobosco, L Lock, S Lock, A Locke, J Locke, T Locke, T Lockett, J Lodge, K Lodhia, M Lofthouse, H Loftus, M Logan, C Logue, SY Loh, S Lokanathan, K Lomme, E London, G Long, N Long, K Longbottom, B Longhurst, M Longshaw, S Longstaffe, J Lonnen, C Lonsdale, L Looby, R Loosley, L Lopes, P Lopez, P Lopez, RW Lord, S Lord, C Lorimer, F Loro, R Lorusso, C Loughlin, W Lovegrove, R Loveless, M Lovell, A Loverdou, A Low, J Low, S Low, A Lowe, C Lowe, E Lowe, F Lowe, L Lowe, M Lowe, R Lowsby, V Lowthorpe, G Lubimbi, A Lubina Solomon, G Lucas, J Lucas, A Lucey, O Lucey, S Luck, LH Lui, A Luintel, H Luke, J Luke, N Lungu, A Lunia, M Lunn, J Luo, M Luscombe, J Luveta, CN Luximon, K Lwin, M Lwin, A Lye, B Lyell, E Lyka, A Lynas, C Lynch, D Lynch, S Lynch, R-G Maamari, H Mabb, L Mabelin, G Mabeza, J Macaro, K Macconaill, C Macdonald, A Macduff, C Macfadyen, JG Macfarlane, J Macfarlane, L Macfarlane, I Macharia, L MacInnes, I MacIntyre, J MacIntyre, K Mack, C Mackay, E Mackay, L Mackay, A Mackenzie, M Mackenzie, R MacKenzie Ross, A Mackey, F Mackie, J Mackie, R Mackie, C Mackinlay, C Mackintosh, K Mackintosh, MJ MacLeod, S Macleod, M Macmahon, A MacNair, C Macphee, I Macpherson, C Macrae, A MacRaild, Y Madani, A Madden, M Madden, C Madden-McKee, S Maddison, N Madeja, P Madhivathanan, M Madhusudhana, A Madu, L Madziva, M Mafham, N Magee, F Magezi, N Maghsoodi, C Magier, LM Magnaye, M Magriplis, M Magtalas, NP Magula, N Mahabir, S Mahadevan-Bava, S Mahajan, A Maharajh, K Maharjan, M Maharjan, A Mahaveer, B Mahay, K Mahay, A Mahdi, H Mahdi, N Mahdi, T Mahendiran, S Mahendran, S Maher, A Maheswaran, S Maheswaran, T Maheswaran, P Mahjoob-Afag, A Mahmood, F Mahmood, H Mahmood, W Mahmood, Z Mahmood, H Mahmoud, M Mahmud, E Mahony, T Mahungu, O Maiga, L Mair, T Majekdunmi, K Majid, A Major, R Major, J Majumdar, MKH Majumder, TLA Mak, A Makan, E Makanju, S Makin, W-O Makinde, Y Makkeyah, ON Makoetlane, M Malanca, H Malcolm, F Malein, N Malhan, A Malicka, A Malik, G Malik, M Maljk, P Mallett, P Mallinder, G Mallison, L Mallon, E Malone, G Maloney, M Mamman, I Man, K Man, R Mancinelli, M Mancuso-Marcello, SK Mandal, S Mandal, T Manders, L Manderson, J Mandeville, T Mane, R Manhas, C Maniero, R Manikonda, I Manjra, R Mankiewitz, B Mann, J Manning, S Manning, P Mannion, K Mansi, K Manso, D Mansour, M Mansour, R Mansour, IT Mapfunde, P Mappa, A Maqsood, H Maraj, C Marchand, N Marcus, A Marcyniuk, M Marecka, D Maren, G Margabanthu, J Margalef, L Margarit, G Margaritopoulos, M Margarson, F Maria del Rocio, T Maria Pfyl, V Mariano, A Maric, G Markham, B Marks, M Marks, P Marks, E Marler, E Marouzet, A Marriott, C Marriott, N Marriott, C Marsden, K Marsden, P Marsden, S Marsden, T Marsden, C Marsh, G Marsh, R Marsh, A Marshall, A Marshall, G Marshall, H Marshall, J Marshall, J Marshall, N Marshall, R Marshall, S Marshall, J Marshall, E Martin, G Martin, H Martin, J Martin, K Martin, L Martin, M Martin, N Martin, T Martin, W Martin, S Martin, T Martindale, M Martineau, L Martinez, JC Martinez Garrido, J Martin-Lazaro, VK Maruthamuthu, B Marwan, G Maryan, R Mary-Genetu, S Maryosh, V Masani, A Mascagni, D Maseda, Z Maseko, S Mashate, Y Mashhoudi, A Mashta, I Masih, S Masih, N Maskell, P Maskell, P Maskey, M Masoli, J Mason, R Mason, C Mason, M Masood, MT Masood, SSME Masood, T Massa, I Massey, J Masters, A Masud, L Matapure, C Matei, R Matewe, E Matey, M Matharu, S Mathen, A Mather, N Mather, J Mathers, J Matheson, A Mathew, A Mathew, M Mathew, V Mathew, J Mathews, K Mathias, A Mathioudakis, S Matibela, D Matila, W Matimba-Mupaya, N Matin, E Matisa, E Matkins, M Matonhodze, E Matovu, J Mattappillil, AJ Matthews, C Matthews, H Matthews, L Mattocks, C Maughan, TT Maulidya, E Mawson, F Maxton, A Maxwell, V Maxwell, E May, J May, P May, I Mayanagao, M Maycock, J Mayer, G Mayers, VA Maynard, K Mayne, T Mayo, L Mayola, S Mayor, I Mazen, T Mazhani, A Mazzella, N Mburu, A Mbuyisha, C Mc Cague, E McAleese, P McAlinden, L McAllister, A McAlpine, G McAlpine, J McAndrew, H McAuley, S McAuliffe, C McBrearty, E McBride, M McBuigan, J McBurney, L McCabe, GL McCafferty, L McCafferty, A McCairn, J McCammon, N McCammon, C McCann, E McCann, A McCarrick, B McCarron, E McCarthy, M McCarthy, N McCarthy, S McCaughey, T McClay, B McClelland, D McClintock, M McCloskey, K McCollum, A McCorkindale, P McCormack, J McCormick, W McCormick, P McCourt, J McCrae, S McCready, G McCreath, H McCreedy, C McCue, IJ McCullagh, L McCullagh, M McCullagh, C McCullough, K McCullough, N McCullough, S McCullough, F McCurrach, J McDermott, P McDermott, R McDermott, K McDevitt, H McDill, B McDonald, C McDonald, D McDonald, R McDonald, S McDonald, D McDonald, N McDonnell, C McDougall, L McDougall, R McDougall, I McEleavy, F McElwaine, J McEntee, E McEvoy, C McEwan, R McEwen, M McFadden, D McFarland, M McFarland, R McFarland, J McFlynn, E McGarry, L McGarvey, A McGeachan, F McGee, L McGenily, C McGettigan, M McGettrick, C McGhee, F McGill, S McGinnity, N McGlinchey, P McGlone, D McGlynn, C McGoldrick, E McGough, C McGovern, R McGovern, A McGowan, A McGown, B McGrath, A McGregor, MP McGuigan, H McGuinness, S McGuire, T McHugh, C McInnes, N McInnes, J McIntosh, K McIntyre, M McIntyre, L McKay, CP McKeag, J McKeane, M McKee, J McKeever, J McKenna, S McKenna, M McKenzie, D McKeogh, C McKerr, AM McKie, H Mckie, L Mckie, G McKnight, H McLachlan, A McLaren, B McLaren, N McLarty, D Mclaughlan, M McLaughlin, J McLay, M McLeish, T McLennan, S McLure, AM McMahon, G McMahon, M McMahon, S McMahon, T McManus, M McMaster, P McMaster, P McMaster, F Mcmeeken, S McMeekin, N McMillan, K McMillen, J McMinn, L McMorrow, H McMullen, C McMurran, H McNally, F McNeela, L McNeil, C McNeill, J McNeill, S McNeill, U McNelis, M McNulty, R McNulty, C McParland, M McPhail, A McQueen, A McSkeane, D McSorland, T McSorley, G McTaggart, J McTaggart, J Mead, P Mead, E Meadows, O Meakin, B Mearns, C Mearns, K Mears, W Mears, M Meda, A Mediana, R Medine, T Medveczky, S Meehan, E Meeks, A Megan, N Meghani, S Meghjee, S Megson, A Mehar, MN Mehmood, R Mehra, R Mehta, G Meintjes, J Meirill, J Meiring, R Mejri, E Mekonnen, S Melander, A-S Melinte, J Mellersh, L Melling, C Mellish, F Mellor, J Mellor, S Mellor, Z Mellor, K Mellows, V Melnic, A Melville, D Melville, J Melville, H Membrey, M Mencias, A Mendelski, M Mendelson, C Mendonca, C Meney, C Menezes, W Mensah, JE Mensshan, A Mentzer, D Menzies, S Menzies, S Mepham, O Mercer, P Mercer, A Merchant, F Merchant, M Mercioniu, M Meredith, M Merida Morillas, B Merrick, J Merritt, S Merritt, P Merron, E Merwaha, S Message, J Messenger, G Metcalf-Cuenca, A Metcalfe, B Metcalfe, K Metcalfe, S Metherell, A Metryka, L Mew, S Meyrick, N Mguni, J Mhlongo, A Miah, J Miah, N Miah, A Mian, G Mic, L Micah-Amuah, D Micallef, A Michael, S Michael, N Michalak, L Michalca-Mason, O Michalec, J Middle, H Middleton, J Middleton, M Middleton, S Middleton, S Mieres, L Mihalca-Mason, T Mikolasch, S Milgate, C Millar, J Millar, J Millard, D Miller, J Miller, L Miller, R Miller, N Miller-Biot, A Miller-Fik, L Millett, B Milligan, H Milligan, I Milligan, C Milliken, K Millington, R Millington, S Millington, H Mills, J Mills, R Mills, H Millward, R Miln, A Milne, C Milne, L Milne, J Milner, L Milner, Z Min, S Mindel, N Minh, PA Minkah, C Minnis, P Minnis, K Minou, N Minskip, J Minton, F Miranda, M Mirela, T Mirza, A Misbahuddin, A Mishra, B Mishra, E Mishra, R Mishra, S Misra, D Mistry, H Mistry, D Mital, S Mitchard, B Mitchell, C Mitchell, LJ Mitchell, P Mitchell, P Mitchelmore, A Mitra, A Mitra, S Mitra, N Mlambo, E Moakes, K Moar, E Moatt, D Mock Font, G Modgil, A Mohamed, A Mohamed, O Mohamed, A Mohammad, W Mohammad, A Mohammed, O Mohammed, YNS Mohammed, B Mohamud, A Moharram, H-P Mok, J Mok, L Mokogwu, M Molina, C Moller-Christensen, M Mollet, M Molloholli, A Molloy, L Molloy, A Molyneux, R Molyneux, T Momoniat, H Monaghan, K Monaghan, S Mongolu, T Monika, K Monsell, M Montasser, A Montgomery, H Montgomery, P Moodley, M Moody, N Moody, A Moon, J Moon, J-H Moon, M Moon, M Moonan, P Moondi, S Moorby, J Moorcroft, A Moore, C Moore, DAJ Moore, F Moore, J Moore, L Moore, N Moore, S Moore, V Moore, R Moores, E Morab, J Morales, N Moramorell, L Moran, G Moray, J Moreno-Cuesta, A Morgan, C Morgan, H Morgan, K Morgan, L Morgan, M Morgan, P Morgan, K Morgan-Jones, E Morgan-Smith, J Morilla, A Morley, T Morley, W Morley, A Morris, D Morris, F Morris, H Morris, J Morris, K Morris, L Morris, M-A Morris, N Morris, P Morris, S Morris, D Morrison, M Morrison, S Morrison, M Morrissey, AC Morrow, A Morrow, F Morselli, G Mortem, V Mortland, C Morton, G Morton, P Morzaria, D Mosby, L Moseley, K Moshal, B Moshy, A Moss, C Moss, J Moss, S Moss, O Mostafa, G Moth, N Motherwell, S Mottershaw, H Moudgil, J Mouland, C Moulds, H Moulton, G Mounce, E Mousley, C Mowatt, K Moxham, B Moya, Q Moyo, E Mshengu, S Mtuwa, A Muazzam, IA Muazzam, N Muchenje, D Mudawi, G Muddegowda, R Mufti, I Mugal, A Mughal, J Muglu, F Muhammad, J Muhammad, C Muir, A Mukherjee, D Mukherjee, J Mukhtar, SAA Mukhtar, D Mukimbiri, J Mulcahy, M Mulcahy, P Mulgrew, B Mulhearn, A Mulla, D Mullan, D Mullasseril Kutten, N Mullen, R Mullett, C Mulligan, S Mulligan, L Mumelj, A Mumford, M Munavvar, H Munby, H Munday, A Munro, S Munt, M Mupudzi, A Murad, OH Muraina, K Muralidhara, M Murdoch, J Murira, A Murphy, B Murphy, C Murphy, E Murphy, G Murphy, H Murphy, P Murphy, R Murphy, S Murphy, C Murray, D Murray, E Murray, K Murray, L Murray, T Murray, E Murtagh, M Murthy, C Murton, R Murton, N Muru, R Musanhu, M Mushabe, O Mushtaq, S Musini, AMM Mustafa, E Mustafa, M Mustafa, I Mustapha, N Mustfa, Z Mustufvi, C Mutch, R Mutch, E Mutema, B Muthukrishnan, S Mutton, N Muzengi, M Mwadeyi, B Mwale, E Mwaura, R Myagerimath, A Myers, S Myers, JS Myerson, K Myint, Y Myint, G Mynott, L Myslivecek, P Nabayego, E Nadar, I Nadeem, M Nadheem, B Nadjm, A Naeem, H Naeem, S Naeem, S Nafees, M Nafei, W Naftalia, T Nagarajan, L Naglik, I Nagra, D Nagra, M Naguib, K Naguleswaran, KS Nagumantry, K Naicker, S Naidoo, V Naidoo, G Naik, R Naik, S Naik, DS Nair, R Nair, T Nair, J Naisbitt, K Naismith, D Nakiboneka-Ssenabulya, S Nallapareddy, S Nallapeta, A Nallasivan, HX Nam, U Nanda, A Nandani, T Nandwani, AR Naqvi, A Naqvi, S Naqvi, S Nasa, D Nash, N Nasheed, A Nasimudeen, U Nasir, N Nasronudin, T Nasser, A Natarajan, G Natarajan, N Natarajan, R Natarajan, P Nath, N Nathaniel, M Nathvani, P Nathwani, G Nava, N Navaneetham, J Navaratnam, H Navarra, S Naveed, J Navin, K Nawaz, S Nawaz, B Nayar, S Naylor, M Nayyar, F Naz, M Naz, B Nazari, A Nazir, S Nazir, D Ncomanzi, O Ndefo, NB Ndoumbe, A Neal, E Neary, M Negmeldin, J Neil, P Neill, HE Neils, A Nejad, J Nel, L Nel, A Nelson, B Nelson, L Nelson, M Nelson, R Nelson, S Nelson, E Nelwan, EJ Nelwan, R Nemane, S Nepal, D Nethercott, K Netherton, K Nettleton, K Neupane, A Newby, D Newby, T Newcombe, H Newell, C Newman, D Newman, H Newman, J Newman, O Newman, T Newman, R Newport, M Newton, AYKC Ng, HEJ Ng, KW Ng, M Ng, S Ng, WJ Ng, YWM Ng, T Ngan, TH Ngo, GCE Ngui, A Ngumo, HK Nguyen, MT Nguyen, N Nguyen, NTT Nguyen, Q Nguyen, THT Nguyen, TT Nguyen, TTN Nguyen, TT Nguyen, TTP Nguyen, K Ngwenya, C Nic Fhogartaigh, N Nicholas, P Nicholas, R Nicholas, D Nicholls, L Nicholls, S Nicholls, A Nicholson, I Nickson, E Nicol, R Nicol, P Nicola, A Nicoll, T Nightingale, F Nikita, P Nikolaos, G Nikonovich, A Nilsson, K Nimako, L Nimako, C Nimmo, P Ninan, T Ninh, M Nirmalan, R Niroula, A Nisar, M Nisar, T Nisar, T Nisbett, A Nisha James, S Nishat, T Nishiyama, S Nix, J Nixon, M Nixon, K Nizam Ud Din, M Nizami, S Nizamis, R Njafuh, I Noakes, L Noba, J Noble, H Noble, HM Noe, J Nolan, J Nolasco, Z Noor, Z Noori, J Norcliffe, L Norman, R Norman, E Norris, K Norris, L Norris, SA Nortcliffe, F North, J North, T North, J Northfield, S Northover, J Nortje, D Norton, R Norton, H Notman, K Nourein, T Novak, N Novas Duarte, C Novis, JA Nowak, KP Nu, M Nugdallah, A Nugent, J Nugent, CW Nugroho, N Numbere, K Nundlall, A Nune, K Nunn, M Nunn, J Nunnick, Y Nupa, F Nur, Z Nurgat, R Nurpeni, A Nuttall, L Nwafor, P Nwajiugo, G Nyamugunduru, L Nyanor, M Nyirenda, K Nyland, D O Rinn, D O Shea, M O Toole, M O'Hara, C O'Hara, L O'Keefe, K O'Reilly, W O'Rourke, C Oakley, N Oakley, S Oakley, B Obale, C Oboh, C O'Brien, J O'Brien, K O'Brien, L O'Brien, N O'Brien, R O'Brien, T O'Brien, E O'Bryan, R Obukofe, C O'Callaghan, L O'Connell, T OConnor, C O'Connor, G O'Connor, M Odam, S Oddie, S Oddy, Y Odedina, K Odedra, S Odelberg, N Odell, O Oderinde, J Odone, L O'Donohoe, C O'Donovan, I Odysseos-Beaumont, S O'Farrell, P Offord, M O'Flaherty, E Ofori, T Ogbara, C Ogilvie, C O'Gorman, I Ogunjembola, O Ogunkeye, U Ohia, S Ojha, S Ojha, O Ojo, F O'kane, M O'Kane, T Okeke, E OKell, A Okines, I Okpala, E Okpo, F Okpoko, M Okubanjo, C Oladipo, L Olaivar, R Olaiya, J Olatujoye, T Old, G Oleszkiewicz, A Oliver, C Oliver, J Oliver, L Oliver, M Oliver, Z Oliver, J Oliver-Commey, NO Olokoto, F Olonipile, O Olufuwa, O Olukoya, A Oluwole-Ojo, L O'Malley, IV Omale, PK Omane-Donkor, M Omar, Z Omar, N Omer, E Omoregie, C O'Neill, L O'Neill, C Ong, O Onuoha, C Onyeagor, CN Oo, Z Oo, HC Ooi, SH Ooi, A Oomatia, A Opata, M Opena, R Oram, C Ord, J Ord, C Oreilly, L Orekoya, D O'Riordan, S O'Riordan, I Orlikowska, A Orme, H Orme, L O'Rourke, C Orr, S Orr, C Orton, A Osadcow, R Osagie, R Osanlou, L Osborne, N Osborne, R Osborne, W Osborne, W Osborne, C Osbourne, J Osei-Bobie, J Osman, W Osman, B Osman, G Osoata, M Ostermann, E O'Sullivan, S O'Sullivan, MA Oteng, N Otey, OK Otite, J Ouyang, R Owen, S Owen, E Owens, C Owoo, Y Owoseni, M Owston, R Oxlade, F Ozdes, J Pack, A Packham, S Packham, P Paczko, G Padden, A Padmakumar, C Page, I Page, J Page, S Page, V Page, J Paget, K Pagett, L Paisley, S Pajak, G Pakou, A Pakozdi, S Pal, S Pal, A Palacios, VB Palagiri Sai, V Palaniappan, P Palanivelu, A Palfreeman, H Palfrey, V Palissery, D Palit, S Pallipparambil Antony, J Palman, A Palmer, H Palmer, J Palmer, L Palmer, R Palmer, A Pambouka, I Pamphlett, D Pan, A Pandey, N Pandian, K Pandya, T Pandya, HR Paneru, A Panes, J Pang, YW Pang, R Pangeni, L Pannell, K Pannu, S Pant, S Panthakalam, CT Pantin, N Pao, H Papaconstantinou, NS Papavarnavas, P Papineni, K Paques, AW Paracha, K Paradowski, V Parambil, S Paranamana, SR Parashar, I Parberry, A Parekh, D Parekh, L Parfitt, H Parfrey, O Parikh, G Parish, J Park, V Parkash, A Parker, B Parker, E Parker, H Parker, J Parker, L Parker, N Parker, S Parker, K Parkin, A Parkinson, M Parkinson, V Parkinson, C Parmar, V Parmar, V Parris, C Parrish, B Parry, HC Parry, S Parslow-Williams, M Parsonage, G Parsons, J Parsons, P Parsons, R Partridge, Z Parvez, K Parvin, L Passby, S Passey, H Passmore, J Pastrana, J Patachako, M Patal, S Patch, A Patel, A Patel, B Patel, D Patel, H Patel, J Patel, K Patel, M Patel, N Patel, P Patel, S Patel, T Patel, Z Patel, V Patel, K Paterson, S Pathak, N Pathan, A Patience, D Patience, B Patil, R Patmore, S Patole, L Paton, A Patrick, G Patrick, J Patrick, S Patten, B Pattenden, C Patterson, J Patterson, L Patterson, M Patterson, R Patterson, M Pattrick, D Paudel, K Paudel, M Paudel, S Paudel, M Paul, S Paul, L Pauls, S Paulus, A Pavely, MJ Pavitt, S Pavord, B Payne, E Payne, M Payne, R Payne, L Peacock, S Peacock, H Peake, J Pearce, R Pearse, A Pearson, D Pearson, H Pearson, K Pearson, S Pearson, SA Pearson, A Peasley, H Peddie, S Peebles, R Peek, A Peer, S Peerbhoy, C Pegg, E Peggie, H Peggie, S Peglar, BH Peirce, M Peirse, C Pelham, A Pemberton, M Penacerrada, A Pender, C Pendlebury, J Pendlebury, R Penfold, C Penman, J Penman, R Penman, J Penner, K Penney, A Pennington, J Penny, J Pepperell, R Percival, A Pereira, R Pereira, C Pereira Dias Alves, I Perera, M Perera, E Perez, J Perez, T Perinpanathan, L Periyasamy, E Perkins, I Pernicova, E Perritt, A Perry, E Perry, M Perry, TM Perumpral, G Pessoa-Amorim, R Petch, L Peter, C Peters, L Peters, M Peters, S Peters, T Peters, A Peterson, R Petersen, L Peto, I Petras, B Petrova, M Petrova, E Petrovics, T Pettigrew, M Pezard-Snell, P Pfeffer, G Phalod, NT Pham, VP Pham, TTH Phan, M Phanish, P Phelan, C Philbey, J Philbin, A Phillips, B Phillips, D Phillips, N Phillips, P Phillips, R Phillips, T Phillips, M Phipps, M Phipps, N Phong, NT Phong, V Phongsathorn, P Phuc, M Phull, HTK Phung, N Phuong, A Phuyal, AK Phyo, MTT PI, S Pick, J Pickard, C Pickering, F Pickering, G Pickering, T Pickett, J Pickles, S Pickstock, B Pickwell-Smith, N Pieniazek, C Piercy, A Pieris, S Pilgrim, PA Pillai, S Pillay, L Pilling, Z Pilsworth, H Pinches, S Pinches, K Pine, MT Pinjala, S Pintus, G Piper, T Pirani, M Pitchford, M Pittman, S Pitts, N Plaatjies, N Platt, R Pleass, M Plowright, L Plummer, C Plumptre, J Pobjoy, T Pogreban, C Poku, S Poku, P Polgarova, R Pollard, L Pollock, O Poluyi, GJ Polwarth, F Pomery, IMF Ponce, P Ponnusamy, S Ponnusamy, A Ponnuswamy, I Ponte Bettencourt dos Reis, S Pooboni, A Poole, L Poole, M Poole, S Poon, T Poonian, J Porteous, M Porteous, D Porter, J Porter, L Porter, R Porter, A Posada, K Postlethwaite, M Potdar, C Pothecary, N Pothina, P Potla, D Potoczna, J Pott, A Potter, J Potter, S Potter, T Potter, E Potton, JB Potts, J Potts, K Potts, B Poudyal, U Poultney, K Poulton, V Poustie, J Powell, N Powell, S Powell, D Power, N Power, S Power, J Poxon, E Poyner, R Poyner, A Prabhu, S Prabowo, V Pradhan, G Pradip, H Prady, R Prananingtias, A Prasad, K Prasad, U Prasad, F Prasanth Raj, S Prasath, N Pratiwi, A Pratley, S Pratt, CB Prayuda, D Preiss, C Prendergast, L Prentice, P Prentice, V Prescott, L Presland, C Prest, S Preston, M Pretorius, N Prevatt, S Prew, A Price, C Price, D Price, E Price, K Price, LJ Price, N Price, V Price, R Price-Eland, A Priest, J Prieto, L Primrose, C Prince, J Prince, L Prince, S Pringle, M Prior-Ong, V Pristopan, K Pritchard, L Pritchard, S Pritchard, V Priyash, A Procter, C Proctor, M Protopapas, R Proudfoot, B Prudon, D Pryor, S Pudi, A Puffett, J Pugh, L Pugh, MT Pugh, N Pugh, R Pugh, V Puisa, E Puji Lestari, S Puliyakkadi, J Pullen, K Punia, S Punnilath Abdulsamad, L Purandare, D Purchase, C Purdue, R Purdy, B Purewal, R Purnell, M Pursell, G Purssord, R Purves, S Purvis, K Puspatriani, D Putensen, SI Putu, B Puvaneswaran, A Puxty, K Puxty, Z Puyrigaud, E Pyart, E Pye, M Pynn, T Qadeer, M Qayum, C Quah, S Quaid, N Quail, C Quamina, K Quang, NN Quang, L Quarm, G Quartermaine, R Quartey, T Quasim, S Quaye, A Quayle, E Quek, S Quenby, P Qui, X Qui, V Quick, J Quigley, J-C Quijano-Campos, J Quindoyos, A Quinn, J Quinn, T Quinn, LJ Quist, Q Quratulain, D Qureshi, E Qureshi, H Qureshi, I Qureshi, K Qureshi, N Qureshi, Q Qurratulain, S Qutab, MS Rabbani, S Rabinowicz, M Raceala, A Rachid, BE Rachman, R Rachman, L Rad, J Radford, L Radford, J Radhakrishnan, H Rafferty, MY Rafiq, S Rafiq, C Rafique, J Rafique, M Rafique, R Ragatha, A Raghunathan, A Raguro, SD Raha, S Rahama, M Rahardjani, K Rahilly, F Rahim, AH Rahimi, HR Rahimi, M Rahman, SU Rahman, S Rahmany, P Rai, S Rai, L Raisova, A Raithatha, A Raj, A Rajagopal, P Rajagopalan, N Rajaiah, K Rajalingam, A Rajasekaran, A Rajasri, B Rajbhandari, S Rajbhandari, T Rajeswaran, J Rajeswary, J Rajkanna, I Rajkumar, G Rajmohan, R Rallan, K Ralston, M Ralston, M Ram, B Ramabhadran, F Ramali, M Ramali, A Ramanan, S Ramanna, M Ramasamy, I Rambe, A Ramchandani, D Ramdin, J Ramirez, M Ramirez, G Ramnarain, A Ramnarine, L Ramos, T Rampling, S Ramraj, J Ramsay, A Ramshaw, A Rana, GF Rana, N Rana, R Rana, A Rand, J Rand, H Randheva, P Ranga, M Rangar, H Rangarajan, S Ranjan, H Rank, P Ranka, R Rankhelawon, A Rankin, A Rao, S Rao, D Rao, AA Rasheed, K Rashid, M Rason, V Raspa, S Rastogi, F Rasul, S Ratcliff, S Ratcliffe, P Rath, S Rath, MI Rather, K Rathod, S Rathore, A Ratnakumar, J Ratoff, D Rattehalli, D Ravaccia, M Raval, P Ravencroft, J Raw, R Raw, M Rawal, SA Rawashdeh, H Rawlins, G Ray, A Raymond-White, D Raynard, H Rayner, N Rayner, A Raynsford, S Razvi, Z Razvi, K Read, S Read, M Reay, A Reddington, A Reddy, H Reddy, H Redfearn, A Redfern-Walsh, I Redknap, N Redman, A Redome, J Redome, A Reed, J Reed, A Rees, C Rees, H Rees, J Rees, M Rees, S Rees, T Rees, E Rees-Jones, F Regan, K Regan, M Regan, S Regan, K Rege, A Regmi, A Rehan, A Rehman, H Rehman, S Rehman, Z Rehman, A Reid, J Reid, S Reid, M Reilly, S Reilly, C Reith, A Reka, A Remegoso, D Rengan, L Renouf, S Renshaw, R Renu Vattekkat, H Reschreiter, M Revels, A Revill, G Rewitzky, S Rey, C Reynard, D Reynish, H Reynolds, P Reynolds, J Rhodes, N Riaz, P Ribeiro, E Rice, M Rice, N Rice, M Rich, A Richards, L Richards, S Richards, C Richardson, E Richardson, F Richardson, J Richardson, M Richardson, N Richardson, J Riches, K Riches, L Richmond, R Richmond, W Ricketts, H Rickman, A Riddell, S Ridgway, M Ridha, C Ridley, P Ridley, G Rieck, L Rigby, M Rigby, D Rigler, S Rijal, N Rika, H Riley, M Riley, P Riley, A Rimainar, ZVP Rimba, D Rimmer, W Rina, R Rintoul, A Riordan, D Ripley, N Rippon, C Rishton, M Riste, D Ritchie, J Ritchie, A Ritchings, P Rivera Ortega, V Rivers, B Rizvi, SAS Rizvi, SHM Rizvi, J Robb, E Robbins, C Roberts, G Roberts, I Roberts, J Roberts, K Roberts, M Roberts, N Roberts, P Roberts, R Roberts, V Roberts, C Robertson, J Robertson, J Robertson, K Robertson, N Robertson, S Robertson, M Robertson, N Robin, C Robinson, E Robinson, G Robinson, H Robinson, J Robinson, K Robinson, L Robinson, M Robinson, N Robinson, R Robinson, S Robinson, A Robinson, S Robson, A Rocca, L Roche, S Roche, N Rodden, A Roddick, E Roddy, J Roddy, M Roderick, A Rodger, F Rodger, M Rodger, M Rodger, A Rodgers, D Rodgers, N Rodgers, P Rodgers, R Rodriguez-Belmonte, N Roe, C Roehr, G Rogers, J Rogers, L Rogers, M Rogers, P Rogers, S Rogers, T Rogers, J Rojkova, KK Roka, S Rokadiya, L Rollins, J Rollo, C Rolls, A Rond-Alliston, C Rook, K Rooney, L Rooney, LP Rosaroso, EJ Rosby, A Rose, S Rose, Z Rose, J Rosier, A Roskilly, GA Ross, I Ross, J Ross, J Rossdale, A Ross-Parker, A Rostron, AN Rosyid, A Rothman, J Rothwell, L Roughley, CA Rourke, K Rowan, N Rowan, S Rowan, A Rowe, N Rowe, L Rowe-Leete, B Rowlands, E Rowlands, M Rowley, S Roy, M Roycroft, A Roynon-Reed, AR Royson, S Rozewicz, A Rudenko, S Rudrakumar, B Rudran, S Ruff, P Rughani, R Rule, S Rundell, E Rushforth, J Rushmer, D Rusk, P Russell, R Russell, C Russo, M Rutgers, K Rutkowski, A Ryan, B Ryan, K Ryan, L Ryan, M Ryan, P Ryan, D Ryan-Wakeling, E Rybka, M Ryder, S Ryder, M Saad, G Saalmink, J Sabale, S Sabaretnam, N Sadiq, E Sadler, A Saffy, B Sage, H Sagoo, S Sagrir, R Saha, S Saha, N Sahdev, S Sahedra, J Sahota, N Said, S Saini, V Saini, B Saint, N Sairam, A Sajid, S Sakthi, H Sakuri, M Saladi, A Salam, A Salberg, E Salciute, G Saleeb, M Saleh, H Salih, L Salih, D Salim, S Salisbury, S Saliu, R Salman, J Salmon, R Salmon, D Salutous, M Sam, S Sam, T Samakomva, R Saman, S Samar, S Saminathan, R Samlal, E Sammons, D Sammut, M Sammut, S Sammut, T Sammut, S Sampath, C Sampson, J Sampson, A Samson, J Samuel, M Samuel, R Samuel, TDL Samuel, Y Samuel, K Samuels, T Samuels, J Samways, M Samyraju, I Sana, V Sanchez, A Sanchez Gonzalez, A Sanda-Gomez, P Sandercock, J Sanders, A Sanderson, T Sanderson, K Sandhu, L Sandhu, S Sandow, V Sandrey, S Sands, L Sanga, H Sangha, J Sanghera, M Sangombe, M Sanju, L Sankaran, F Santos, C Santos Ferreira De Almeida, R Santosh, J Sanyal, AF Sanz-Cepero, Y Sapkota, D Saragih, D Saralaya, A Saraswati, A Saraswatula, P Saravanamuthu, S Sarawade, J Sarella, A Sarfatti, R Sargent, B Sari, D Sari, D Sarkar, K Sarkar, R Sarkar, S Sarma, P Sarmiento, Z Sarwar, T Sass, K Satchithananthasivam, S Sathe, S Sathianandan, A Sathyanarayanan, SJP Sathyanarayanan, T Sathyapalan, P Satodia, V Saulite, A Saunders, R Saunders, S Saunders, A Saunderson, H Savill, K Savlani, G Saxena, M Saxton, A Sayan, I Sayers, D Scaletta, D Scanlon, J Scanlon, L Scarratt, S Scattergood, A Schadenberg, J Schafers, W Schneblen, E Schofield, R Schofield, S Schofield, D Scholes, K Scholes, A Schoolmeesters, N Schumacher, N Schunke, M Schuster Bruce, K Schwarz, A Scobie, T Scoones, T Scorrer, A Scott, A Scott, C Scott, E Scott, K Scott, L Scott, M Scott, S Scott, T Scott, Z Scott, S Scourfield, W Scrase, NA Scriven, A Scullion, T Scullion, E Seager, C Seagrave, R Seaman, E Sear, I Seaton, A Seatter, A Seckington, J Sedano, G Seddon, G Sedgwick, Y See, MA Seelarbokus, C Sefton, M Segovia, F Seidu, G Sekadde, F Selby, G Selby, C Sellar, R Sellars, K Sellers, J Selley, V Sellick, G Selvadurai, B Selvarajah, H Selvaskandan, SS Selvendran, J Selwyn, A Semmens, G Semple, M Sen, N Sen, S Sen, A Sengupta, N Sengupta, S Senra, H Senya, T Serafimova, E Sernicola, D Sethi, S Sethi, N Setty, A Seward, T Sewdin, T-A Sewell, J Seymour, K Seymour, H Shabbir, F Shackley, T Shafi, F Shafique, A Shah, A Shah, B Shah, H-A Shah, M Shah, N Shah, P Shah, Q Shah, R Shah, S Shah, SH Shah, W Shah, S Shahad, S Shahi, S Shahnazari, N Shahzad, M Shahzeb, A Shaibu, Z Shaida, AY Shaikh, M Shaikh, R Shail, M Shaji, M Shakeel, R Shakya, K Shalan, M Shameem, N Shamim, U Shamji, A Shams, K Shams, R Shamsah, T Shanahan, H Sharaf, A Sharif, A Sharma, B Sharma, M Sharma, O Sharma, P Sharma, R Sharma, S Sharma, SD Sharma, S Sharma, S Sharma, A Sharp, C Sharp, G Sharp, K Sharp, LM Sharp, P Sharratt, K Sharrocks, S Shashaa, A Shaw, C Shaw, D Shaw, J Shaw, L Shaw, M Shaw, TG Shaw, A Shawcross, J Shawcross, J Shawe, L Shayler, S Shedwell, J Sheffield, Z Shehata, A Sheik, A Sheikh, N Sheikh, B Shelley, S Shelton, A Shenoy, J Shenton, S Shephardson, A Shepherd, K Shepherd, L Shepherd, S Shepherd, G Sheppard, R Sheppeard, H Sheridan, R Sheridan, S Sherridan, L Sherris, S Sherwin, S Shibly, FF Shiham, C Shilladay, B Shillitoe, D Shingadia, C Shioi, A Shirgaonkar, K Shirley, H Shirt, A Shonubi, J Shoote, R Shorrocks, R Shortman, R Shotton, S Shotton, C Shovelton, E Shpuza, A Shrestha, A Shrestha, N Shrestha, R Shrestha, S Shrestha, K Shuker, J Shurlock, J Shurmer, ER Shuvo, SK Siabi, G Siame, L Siamia, M Siaw-Frimpong, S Siddavaram, N Siddique, S Siddique, S Siddique, E Siddle, E Sidebotham, J Sidebottom, R Sievers, K Siggens, N Sikondari, I Silanas, SV Silva, C Silva Moniz, M Sim, T Simangan, V Simbi, R Sime, G Simmons, O Simmons, R Simms, L Simon, M Simon, N Simon, S Simpkins, A Simpson, A Simpson, D Simpson, G Simpson, J Simpson, K Simpson, M Simpson, P Simpson, T Simpson, K Simpson, S Sinclair, C Sing, A Singh, C Singh, D Singh, J Singh, L Singh, M Singh, N Singh, P Singh, S Singh, P Singhal, B Singizi, V Singler, M Sinha, P Sinha, S Sinha, U Sinha, G Sisson, S Sithiravel, K Sivakumar, S Sivakumar, D Sivakumran, S Sivanadarajah, P-R Sivasothy, A Skaria, N Skehan, R Skelly, O Skelton, I Skene, D Skinner, T Skinner, V Skinner, A Skorko, I Skorupinska, M Skorupinska, A Slack, K Slack, H Slade, M Slade, L Slater, N Slawson, R Slingsby, A Sloan, B Sloan, D Sloan, G Sloane, M Slowinska, B Small, E Small, S Small, A Smallridge, D Smalls, KD Smallshaw, A Smallwood, B Smart, L Smart, J Smeaton, C Smit, A Smith, C Smith, D Smith, E Smith, H Smith, I Smith, J Smith, K Smith, L Smith, M Smith, MA Smith, N Smith, O Smith, P Smith, R Smith, S Smith, T Smith, V Smith, S Smolen, S Smuts, N Smyth, A Snell, D Snell, L Snell, A So, B So, M Soan, RF Sobama, T Sobande, S Sobowiec Kouman, A Sobrino Diaz, B Sohail, H Sohal, R Soiza, O Solademi, B Soleimani, A Solesbury, M Soliman, B Solis, R Solly, L Solomon, S Somalanka, C Somashekar, S Sommerfield, G Soni, R Sonia, T Sonoiki, S-C Soo, P Soor, G Soothill, J Soren, A Sothinathan, P Sothirajah, J Sousa, N Soussi, D Southam, D Southern, I Southern, L Southern, SM Southin, J Southwell, T Southworth, S Sowden, J Sowter, C Spalding, E Spata, C Speare, K Spears, M Spears, L Speirs, S Speirs, M Spence, N Spence, B Spencer, G Spencer, R Spencer, S Spencer, T Spencer, H Spickett, J Spillane, W Spiller, K Spinks, M Spinks, N Spittle, S Spray, J Spriggs, O Spring, G Squires, J Squires, R Squires, R Sreenivasan, S Sreenivasan, M Sri, K Sri Paranthamen, R Srikantaiah, K Srinivasan, R Srinivasan, A Srirajamadhuveeti, V Srirathan, SK Ssiabi, R Stacey, S Stacpoole, L Stadon, WJ Stagg, J Staines, N Staines, K Stammers, R Stanciu, G Stanczuk, T Standley, B Staniforth, A Stanton, L Stanton, R Staples, S Stapley, N Staplin, A Stark, E Starkey, DS Starnes, M Starr, R Stead, C Stebbing, C Steele, H Steer, J Steer, V Stefania, P Stefanowska, F Steffensen, C Stemp, E Stenson, A Stephens, D Stephensen, E Stephenson, M Sterrenburg, J Stevens, M Stevens, W Stevens, A Stevenson, L Stevenson, S Stevenson, M Steward, C Stewart, DA Stewart, K Stewart, M Stewart, R Stewart, J Stickley, G Stiller, S Stirrup, S Stock, A Stockdale, D Stocker, L Stockham, P Stockton, E Stoddard, K Stoffberg, C Stokes, B Stone, R Stone, S Stone, E-J Stoner, I Storey, K Storton, F Stourton, A Strachan, C Strait, E Stratton, J Stratton, S Straw, D Streit, E Stride, S Stringer, S Strong-Sheldrake, S Struik, C Stuart, A Stubbs, H Stubbs, A Sturdy, S Sturney, M Stuttard, C Suarez, K Subba, CP Subbe, K Subramaniam, M Subramanian, V Subramanian, C Subudhi, R Suckling, S Sudershan, P Sugden, PA Suherman, R Sukla, A Sukumaran, E Suleiman, A Suliman, F Suliman, S Sultan, U Sumardi, S Sundar, R Sundaram, R Sundhar, E Sung, N Sunni, J Suntharalingam, A Sur, D Suresh, N Suresh, S Suresh, M Surtees, C Susan, D Suter, R Suthar, H Sutherland, R Sutherland, S Sutherland, D Sutinyte, D Sutton, S Sutton, M Sutu, M-L Svensson, S Svirpliene, A Swain, R Swain, T Swaine, C Swales, C Swanson-Low, T Swart, S Sweetman, E Swift, P Swift, R Swift, R Swingler, S Swinhoe, K Swist-Szulik, L Swithenbank, O Syed, C Sykes, D Sykes, E Sykes, L Sylvester, D Symington, D Symon, A Syndercombe, Z Syrimi, J Syson, G Szabo, D Szabó, T Szakmany, N Szarazova, M Szekely, A Szekeres, M Szeto, K Szymiczek, M Tabish, M Tadros, A Tageldin, L Tague, H Tahir, M Tahir, M Tai, J Tait, A Takyi, P Talbot, A Talbot -Smith, J Talbot-Ponsonby, R Tallent, B Tallon, A Talukdar, A Tan, BT Tan, H Tan, J Tan, JS Tan, K Tan, WT Tan, A Tana, A Tanner, C Tanney, T Tanqueray, E Tanton, A Tantri, T Tanzil-Al-Imran, H Tarft, P Taribagil, O Tarin, S Tariq, D Tarpey, E Tarr, L Tarrant, A Tasiou, A Tate, M Tate, ML Tate, P Tate, K Tatham, SS Tavares, V Tavoukjian, SAI Tay, A Taylor, B Taylor, C Taylor, CA Taylor, D Taylor, E Taylor, H Taylor, J Taylor, K Taylor, L Taylor, M Taylor, N Taylor, R Taylor, S Taylor, T Taylor, V Taylor, M Taylor-Siddons, T Taynton, A Te, F Teasdale, J Teasdale, K Teasdale, J Tebbutt, C Tee, I Teeluck, B Tejero Moya, R Tejwani, A Telfer, V Teli, J Tempany, J Temple, N Temple, H Tench, YH Teoh, R Tereszkowski-Kaminski, L Terrett, L Terry, TIM Tesha, D Tetla, S Tewari, D Tewkesbury, J Texeira, C Tey, M Thake, C Thakker, M Thakker, J Thakrar, BJ Thakuri, B Thamu, H Thao, NT Thao, A Thapa, H Thatcher, A Thayanandan, K Thazhatheyil, E Thein, L Theocharidou, P Thet, K Thevarajah, M Thevendra, D Thien, N Thiri Phoo, Y Thirlwall, M Thirumaran, A Thomas, C Thomas, E Thomas, H Thomas, J Thomas, JL Thomas, K Thomas, L Thomas, R Thomas, S Thomas, T Thomas, V Thomas, K Thomasson, R Thomas-Turner, C Thompson, E Thompson, F Thompson, H Thompson, J Thompson, K Thompson, L Thompson, M Thompson, O Thompson, R Thompson, Y Thompson, BG Thomson, N Thomson, P Thorburn, N Thorn, C Thorne, N Thorne, A Thornton, D Thornton, J Thornton, R Thornton, S Thornton, T Thornton, C Thorpe, N Thorpe, S Thorpe, P Thozthumparambil, L Thrasyvoulou, H Thraves, N Thu, G Thueux, NTH Thuong, P Thu-Ta, D Thuy, V Thwaiotes, CL Thwaites, G Thwaites, S Tiberi, S Tieger, C Tierney, M Tighe, S Tilbey, C Till, A Tiller, H Tiller, J Timerick, E Timlick, A Timmins, A Timmis, H Timms, A-M Timoroksa, S Tinashe, S Tingley, N Tinker, H Tinkler, M Tinkler, J Tipper, A Tirumalai Adisesh, H Tivenan, K Tluchowska, H T-Michael, A Todd, J Todd, S Todd, O Toffoletti, M Tohfa, S Tohill, M Tolson, A Tomas, N Tomasova, S Tomlin, S Tomlins, J Tomlinson, K Tomlinson, J Tonkin, I Tonna, C Toohey, K Topham, M Topping, A Torokwa, C Torrance, O Touma, L Tous Sampol, R Tousis, M Tout, P Tovey, G Towersey, J Townley, R Tozer, DK Tran, H Tran, HB Tran, M Tran, NB Tran, VG Tran, VK Tran, H Tranter, J Travers, C Travill, S Traynor, L Trethowan, E Treus Gude, M Trevelyan, NA Trewick, A Tridente, H Trieu, S Triggs, F Trim, A Trimmings, T Trinick, S Tripathy, K Trivedi, S Troedson, E Tropman, A Trotter, S Trous, H Trower, M Trowsdale Stannard, N Trudgill, R Truell, N Truman, M Truslove, S Trussell, T Trussell, K Tsakiridou, C Tsang, P Tsang, T Tsawayo, KK Tsilimpari, G Tsinaslanidis, M Tsitsi, S Tso, N Tucker, S Tucker, S Tucker, DE Tudor, A Tufail, J Tuff, J Tuffney, R Tully, T Tulus Satriasih, G Tunesi, D Tung, K Turbitt, R Turel, T Turgut, C Turley, A Turnbull, A Turner, C Turner, G Turner, K Turner, L Turner, LC Turner, M Turner, P Turner, S Turner, V Turner, I Turner-bone, S Turney, J Turvey, C Tweed, D Tweed, R Twemlow, E Twohey, B Tyagi, V Tyagi, A Tyer, A Tyler, J Tyler, A Tyzack, P Tzavaras, I Tzinieris, AW Uddin, MS Uddin, R Uddin, J Ugoji, E Ukaegbu, M Ul Haq, W Ul Hassan, Z Ul-Haq, S Ullah, J Um, A Umaipalan, A Umate, J Umeadi, A Umeh, W Umeojiako, B Ummat, E Underhill, C Underwood, J Underwood, A Unsworth, V Uppal, VS Uppal, G Upson, M Ur Rasool, A Uriel, S Urruela, H Uru, J Usher, M Usher, R Usher, A Usher-Rea, A Ustianowski, E Usuf, FN Utomo, H Uzu, LC Vaccari, U Vaghela, A Vaidya, D Vail, B Valecka, J Valentine, B Valeria, P Vallabhaneni, T Valleri, N Vallotton, L Vamplew, E Vamvakiti, J Vamvakopoulos, S Van Blydenstein, L van Bruggen, M van de Venne, A van der Meer, N van der Stelt, R Van Doorn, L van Koutrik, A Van Loggerenberg, J Vance-Daniel, R Vancheeswaran, SI Vandeyoon, P Vankayalapati, P Vanmali, C Vansomeren, W Van't Hoff, KN Vanya, S Vara, SJ Vardy, A Varghese, M Varghese, W Varney, G Varnier, A-N Varouxaki, R Varquez, V Vasadi, O Vass, K Vassell, V Vasu, V Vasudevan, M Vatish, S Vaughan, H Vayalaman, D Vayapooree, C Vaz, N Veale, S Veerasamy, S Velankar, L Velauthar, N Veli, N Vella, A Velugupati, A Velusamy, I Venables, M Venditti, R Venkataramakrishnan, R Venn, M Venter, L Ventilacion, J Vere, M Veres, S Vergnano, W Verling, A Verma, R Vernall, B Vernon, M Vertue, L Verueco, J Verula, A Veterini, N Vethanayagam, S Vettikumaran, L Veys, C Vickers, S Victor, S Victoria, CP Vidaillac, J Vidler, B Vijayakumar, VW Vijayaraghavan Nalini, B Vilcinskaite, A Vileito, N Vilimiene, L Vinall, S Vinay, L Vinayakarao, O Vincent, R Vincent, P Virdee, E Virgilio, AM Virk, E Visentin, M Vitaglione, K Vithian, S Vittoria, S Vivekananthan, E Vlad, B Vlies, L von Oven, C Vooght, KT Vu Thai, K Vutipongsatorn, A Vuylsteke, E Vyras, R Wach, B Wadams, S Wadd, N Waddington, P Wade, J Wadsley, K Wadsworth, SEI Wafa, D Wagstaff, L Wagstaff, D Wahab, Z Wahbi, A Waheed Adigun, S Waidyanatha, A Waite, R Wake, A Wakefield, W Wakeford, F Wakinshaw, E Waldeck, A Walden, L Walding, A Waldron, J Waldron, E Wales, B Wali, D Walker, G Walker, H Walker, I Walker, K Walker, L Walker, O Walker, R Walker, S Walker, G Wallace, R Wallbutton, J Wallen, K Wallendszus, A Waller, R Waller, G Wallis, L Wallis, M Wallis, E Walmsley, D Walsh, E Walsh, L Walsh, D Walstow, D Walter, A Walters, H Walters, J Walters, E Walton, L Walton, M Walton, O Walton, S Walton, M Wan, J Wanda, M Wands, R Wane, F Wang, N Wang, R Wang, S Wang, D Warbrick, S Warburton, C Ward, D Ward, E Ward, H Ward, J Ward, L Ward, N Ward, R Ward, T Ward, SA Warden, G Wardere, S Wardle, H Wardy, G Waring, S Waring, J Warmington, B Warner, C Warner, L Warnock, S Warran, J Warren, L Warren, R Warren, Y Warren, D Warrender, H Warren-Miell, A Warris, G Warwick, H Wassall, S Wasserman, E Wasson, HJ Watchorn, H Waterfall, A Waters, D Waters, M Waterstone, A Watkin, C Watkins, E Watkins, K Watkins, L Watkins, A Watson, AJR Watson, E Watson, F Watson, JGR Watson, L Watson, P Watson, R Watson, K Watson, M Watters, D Watterson, K Wattimena, D Watts, J Watts, M Watts, V Waugh, E Wayman, M Wayman, A Wazir, M Weatherhead, N Weatherly, C Webb, H Webb, K Webb, S Webb, C Websdale, D Webster, I Webster, J Webster, T Webster, J Wedlin, L Wee, R Weerakoon, T Weerasinghe, J Weeratunga, M Weetman, S Wei, I Weichert, E Welch, H Welch, J Welch, L Welch, S Welch, B Welham, S Weller, L Wellings, B Wells, S Wellstead, B Welsh, R Welsh, I Welters, R Welton, V Wenn, L Wentworth, J Wesonga, K Wesseldine, J West, M West, R West, S West, L Western, R Westhead, H Weston, A Westwood, K Westwood, S Westwood, B Wetherill, S Wheaver, H Wheeler, B Whelan, M Whelband, A Whileman, A Whitcher, A White, B White, C White, D White, J White, K White, M White, N White, S White, T White, C Whitehead, K Whitehorn, A Whitehouse, C Whitehouse, T Whitehouse, J Whiteley, L Whiteley, S Whiteley, R Whitham, G Whitlingum, D Whitmore, E Whittaker, L Whittam, A Whittington, H Whittle, R Whittle, E Wiafe, L Wiblin, O Wickens, J Widdrington, J Wieboldt, H Wieringa, C Wiesender, L Wiffen, A Wight, A Wignall, C Wignall, A Wilce, D Wilcock, E Wilcock, L Wilcox, B Wild, L Wild, S Wild, M Wilde, L Wilding, P Wilding, T Wildsmith, J Wileman, J Wiles, K Wiles, E Wilhelmsen, T Wiliams, J Wilkie, D Wilkin, H Wilkins, J Wilkins, S Wilkins, I Wilkinson, L Wilkinson, N Wilkinson, S Wilkinson, T Wilkinson, S Willetts, A Williams, C Williams, CV Williams, D Williams, E Williams, G Williams, H Williams, J Williams, K Williams, M Williams, P Williams, R Williams, S Williams, T Williams, S Williams, A Williamson, C Williamson, D Williamson, J Williamson, JD Williamson, R Williamson, C Williamson, H Williamson, E Willis, H Willis, J Willis, L Wills, L Willsher, C Willshire, F Willson, J Willson, A Wilson, B Wilson, D Wilson, I Wilson, J Wilson, K Wilson, K-A Wilson, L Wilson, M Wilson, S Wilson, T Wilson, J Wilson, KLY Win, M Win, T Win, TT Win, WYW Win, L Winckworth, L Winder, P Winder, S Winearl, H Winmill, S Winn, C Winpenny, H Winslow, H Winter, J Winter, B Winter-Goodwin, J Winterton, H Winwood, J Wischhusen, S Wisdom, M Wise, M Wiselka, R Wiseman, S Wiseman, S Wishart, T WIshlade, E Witele, N Withers, J Wittes, D Wixted, T Wodehouse, W Wolf, N Wolff, K Wolffsohn, R Wolf-Roberts, E Wolodimeroff, A Wolstencroft, A Wong, C Wong, C-H Wong, C-M Wong, E Wong, JSY Wong, KY Wong, MY Wong, N Wong, S Wong, T Wong, AA Wongkyezeng, A Wood, C Wood, D Wood, F Wood, G Wood, H Wood, J Wood, L Wood, M Wood, S Wood, T Wood, K Woodall, R Woodfield, C Woodford, E Woodford, J Woodford, L Woodhead, T Woodhead, P Woodland, M Woodman, S Woodmansey, C Woods, J Woods, K Woods, S Woods, Z Woodward, M Woolcock, G Wooldridge, R Woolf, C Woollard, L Woollen, E Woolley, J Woolley, D Woosey, D Wootton, J Wootton, D Worley, S Worton, J Wraight, M Wray, K Wren, L Wren, C Wrey Brown, C Wright, D Wright, F Wright, H Wright, I Wright, L Wright, R Wright, S Wright, T Wright, C Wroe, H Wroe, H Wu, P Wu, J Wubetu, F Wulandari, R Wulandari, S Wurie, C Wyatt, F Wyn-Griffiths, I Wynter, B Xavier, A Xhikola, BE Xia, Z Xia, E Yacoba, S Yadav, M Yakubi, M Yan, Y Yanagisawa, F Yang, Y Yang, M Yanney, WL Yap, N Yaqoob, S Yasmin, B Yates, D Yates, E Yates, H Yates, T Yates, M Yates, J Ye, C Yearwood Martin, K Yein, F Yelnoorkar, LM Yen, A Yeoh, CY Yeung, P Yew, D Yewatkar, L Ylquimiche Melly, I Ynter, H Yong, J Yorke, J Youens, A Younes Ibrahim, E Young, G Young, L Young, A Yousafzar, S Youssouf, A Yousuf, H Yovita, C Yu, JSJ Yuan, N Yufaniaputri, B Yung, D Yusef, S Yusef, I Yusuf, A-S Zafar, S Zagalo, S Zaher, A Zahoor, M Zainab, T Zak, K Zaki, N Zakir, K Zalewska, A Zamalloa, M Zaman, S Zaman, J Zamikula, L Zammit, M Zammit-Mangion, M Zawadzka, M Zayed, E Zebracki, D Zehnder, L Zeidan, D Zeinali, J Zhang, X Zhao, D Zheng, D Zhu, M Zia, O Zibdeh, R Zill-E-Huma, ET Zin, E Zincone, G Zindoga, E Zinkin, V Zinyemba, C Zipitis, L Zitter, A Zmierczak, G Zubikarai, A Zubir, N Zuhra, R Zulaikha, S Zulfikar, C Zullo, A Zuriaga-Alvaro

## Abstract

**Background:**

Empagliflozin has been proposed as a treatment for COVID-19 on the basis of its anti-inflammatory, metabolic, and haemodynamic effects. The RECOVERY trial aimed to assess its safety and efficacy in patients admitted to hospital with COVID-19.

**Methods:**

In the randomised, controlled, open-label RECOVERY trial, several possible treatments are compared with usual care in patients hospitalised with COVID-19. In this analysis, we assess eligible and consenting adults who were randomly allocated in a 1:1 ratio to either usual standard of care alone or usual standard of care plus oral empagliflozin 10 mg once daily for 28 days or until discharge (whichever came first) using web-based simple (unstratified) randomisation with allocation concealment. The primary outcome was 28-day mortality; secondary outcomes were duration of hospitalisation and (among participants not on invasive mechanical ventilation at baseline) the composite of invasive mechanical ventilation or death. On March 3, 2023 the independent data monitoring committee recommended that the investigators review the data and recruitment was consequently stopped on March 7, 2023. The ongoing RECOVERY trial is registered with ISRCTN (50189673) and ClinicalTrials.gov (NCT04381936).

**Findings:**

Between July 28, 2021 and March 6, 2023, 4271 patients were randomly allocated to receive either empagliflozin (2113 patients) or usual care alone (2158 patients). Primary and secondary outcome data were known for greater than 99% of randomly assigned patients. Overall, 289 (14%) of 2113 patients allocated to empagliflozin and 307 (14%) of 2158 patients allocated to usual care died within 28 days (rate ratio 0·96 [95% CI 0·82–1·13]; p=0·64). There was no evidence of significant differences in duration of hospitalisation (median 8 days for both groups) or the proportion of patients discharged from hospital alive within 28 days (1678 [79%] in the empagliflozin group *vs* 1677 [78%] in the usual care group; rate ratio 1·03 [95% CI 0·96–1·10]; p=0·44). Among those not on invasive mechanical ventilation at baseline, there was no evidence of a significant difference in the proportion meeting the composite endpoint of invasive mechanical ventilation or death (338 [16%] of 2084 *vs* 371 [17%] of 2143; risk ratio 0·95 [95% CI 0·84–1·08]; p=0·44). Two serious adverse events believed to be related to empagliflozin were reported: both were ketosis without acidosis.

**Interpretation:**

In adults hospitalised with COVID-19, empagliflozin was not associated with reductions in 28-day mortality, duration of hospital stay, or risk of progressing to invasive mechanical ventilation or death so is not indicated for the treatment of such patients unless there is an established indication due to a different condition such as diabetes.

**Funding:**

UK Research and Innovation (Medical Research Council) and National Institute of Health Research (MC_PC_19056), Wellcome Trust (222406/Z/20/Z), and Foreign, Commonwealth and Development Office (Project Number 204765-112).

**Translations:**

For the Nepali, Hindi, Indonesian (Bahasa) and Vietnamese translations of the abstract see Supplementary Materials section.

## Introduction

Patients with cardiometabolic diseases (such as heart failure, diabetes, and chronic kidney disease) are at increased risk of hospitalisation and death from COVID-19.^1^ SGLT2 inhibitors have been shown to reduce cardiovascular and kidney events in patients with cardiometabolic diseases.^2^ The precise mechanisms underlying such benefits are not known, but SGLT2 inhibitors appear to favourably modify some pathways that are dysregulated in acute illnesses such as COVID-19.

Inflammation is a key feature of severe COVID-19. Markedly raised levels of inflammatory markers such as C-reactive protein, ferritin, interleukin-6 (IL-6), and other cytokines are observed in severe cases and are associated with poor outcomes.^3^, ^4^ Corticosteroids, IL-6 inhibitors, and Janus kinase (JAK) inhibitors have been shown to reduce mortality in patients with severe COVID-19.^5^, ^6^, ^7^ Together, these results show that inflammation is modifiable and anti-inflammatory therapy can improve clinical outcomes. SGLT2 inhibitors can reduce inflammation,^8^, ^9^ via different mechanisms including attenuation of the nucleotide binding domain-like pyrin domain 3 (NLRP3) inflammasome, which correlates with disease severity in COVID-19.^10^, ^11^ A meta-analysis of 26 trials in patients with type 2 diabetes showed a reduced risk of pneumonia and septic shock among those receiving SGLT2 inhibitors.^12^ In addition, SGLT2 inhibitors inhibit glycolysis and stimulate lipolysis, which could create a less favourable energetic environment for viruses such as SARS-CoV-2,^13^, ^14^, ^15^ and improve endothelial function.^16^

**Research in context**

**Evidence before this study**

We searched Medline, Embase, MedRxiv and the WHO International Clinical Trials Registry Platform between Sept 1, 2019, and March 13, 2023 for randomised controlled trials comparing the effect of SGLT2 inhibitors and usual care or placebo in patients hospitalised with COVID-19 using the search terms: (Coronavirus Infections/ or coronavirus infection$.mp. or SARS-COV-2.mp. or SARS-CoV-2/ or Coronavirus/ or Coronavirus$.mp. or Covid.mp. or Covid-19.mp or COVID-19/ or 2019n-CoV.mp. or covid19.mp or SARSCoV2.mp. or SARS-Cov2.mp.) AND (Sodium-Glucose Transport 2 Inhibitors/ or (sglt2 or sglt-2 or sglt 2).mp. or (SGLT-2 inhibitor$ or SGLT2 inhibitor$ or SGLT 2 inhibitor$).mp. or (sodium-glucose transporter$ or sodium glucose transporter$.mp.) or (sodium glucose co?transporter$ or sodium-glucose co?transporter$).mp. or (canagliflozin$ or dapagliflozin$ or empagliflozin$ or ertugliflozin$ or ipragliflozin$ or luseogliflozin$ or remogliflozin$ or sergliflozin$ or sotagliflozin$ or tofogliflozin$ or bexagliflozin$).mp.) and using validated filters to select for randomised controlled trials. No language restrictions were applied.

We identified one trial (DARE-19) with results available that assessed an SGLT2 inhibitor in hospitalised COVID-19 patients. This trial was at low risk of bias and found a non-significant reduction in mortality with SGLT2 inhibitors, but with only 95 deaths was not large enough to exclude a clinically important effect.

**Added value of this study**

To our knowledge, the Randomised Evaluation of COVID-19 Therapy (RECOVERY) trial is the largest randomised trial of the effect of SGLT2 inhibitors in patients hospitalised with COVID-19 and included patients from three continents. We found no evidence of benefit. Overall, 289 (14%) of 2113 patients allocated to empagliflozin and 307 (14%) of 2158 patients allocated to usual care died within 28 days (rate ratio 0·96 [95% CI 0·82–1·13]; p=0·64).

**Implications of all the available evidence**

There is no good evidence that treatment with an SGLT2 inhibitor is of clinical benefit for adults hospitalised with COVID-19 in addition to current usual care.

The DARE-19 trial compared the SGLT2 inhibitor dapagliflozin 10 mg once daily with placebo in 1250 patients hospitalised with COVID-19 who had at least one cardiometabolic risk factor (with or without diabetes) but were not critically ill.^17^ The primary outcome of new or worsened organ dysfunction or death occurred in 70 (11%) of 625 participants in the dapaglifozin group versus 86 (14%) of 625 in the placebo group (hazard ratio [HR] 0·80, 95% CI 0·58–1·10). The dual primary outcome of improvement in clinical status by day 30 was also not significantly affected (win ratio 1·09, 95% CI 0·97–1·22). Dapagliflozin was well-tolerated and appeared safe (with fewer serious adverse events reported in the dapaglifozin group than placebo). Here, we aimed to assess the safety and efficacy of another SGLT-2 inhibitor, empagliflozin, in patients admitted to hospital with COVID-19.

## Methods

### Study design and participants

The Randomised Evaluation of COVID-19 therapy (RECOVERY) trial is an investigator-initiated, individually randomised, controlled, open-label, adaptive platform trial to evaluate the effects of potential treatments in patients hospitalised with COVID-19. Details of the trial design and results for other possible treatments (dexamethasone, hydroxychloroquine, lopinavir–ritonavir, azithromycin, tocilizumab, convalescent plasma, colchicine, aspirin, casirivimab plus imdevimab, baricitinib, and high-dose corticosteroids in hypoxic patients not requiring ventilatory support) have been published previously.^7^, ^18^, ^19^, ^20^, ^21^, ^22^, ^23^, ^24^, ^25^, ^26^, ^27^ The trial is underway at hospital organisations in the UK supported by the National Institute for Health and Care Research Clinical Research Network, as well as in south and southeast Asia and Africa. Of these, 118 hospitals in the UK, five in Nepal, four in Indonesia, two in Viet Nam, four in South Africa, one in Ghana, and five in India enrolled participants in the evaluation of empagliflozin (appendix 5 pp 2–31). The trial is coordinated by the Nuffield Department of Population Health at University of Oxford (Oxford, UK), the trial sponsor. The trial is conducted in accordance with the principles of the International Conference on Harmonisation Good Clinical Practice guidelines and approved by the UK Medicines and Healthcare products Regulatory Agency and the Cambridge East Research Ethics Committee (20/EE/0101). The protocol, statistical analysis plan, and additional information are available online.

Patients admitted to hospital were eligible for the study if they had clinically suspected or laboratory confirmed SARS-CoV-2 infection and no medical history that might, in the opinion of the attending clinician, put the patient at significant risk if they were to participate in the trial. Children (aged <18 years) and pregnant women were not eligible for randomisation due to limited data on use of empagliflozin in these groups such that it was not possible to make an evidence-based benefit-risk assessment. Patients with type 1 diabetes (or post-pancreatectomy diabetes), a history of ketoacidosis, or type 2 diabetes with ketosis at the time of recruitment were ineligible for the comparison of empagliflozin versus usual care (further details in appendix 5 pp 32–33). Written informed consent was obtained from all patients, or a legal representative if patients were too unwell or unable to provide consent.

### Randomisation and masking

Baseline data were collected using a web-based case report form that included demographics, level of respiratory support, major comorbidities, suitability of the study treatment for a particular patient, SARS-CoV-2 vaccination status, and treatment availability at the study site (appendix 5 pp 43–45). For some patients, empagliflozin was unavailable at the hospital at the time of enrolment or was considered by the managing physician to be either definitely indicated or definitely contraindicated. These patients were excluded from the randomised comparison between empagliflozin and usual care. Eligible and consenting adult patients were assigned in a 1:1 ratio to either usual standard of care or usual standard of care plus empagliflozin using web-based simple (unstratified) randomisation with allocation concealed until after randomisation (appendix 5 pp 41–43). Investigators were instructed to monitor blood ketones twice daily (or urine ketones once daily if testing was not available in the vicinity of the patient) and how to manage ketosis if it occurred during treatment with empagliflozin (appendix 5 pp 32–33).

As a platform trial, and in a factorial design, patients could be simultaneously randomly assigned to other treatment groups: baricitinib versus usual care, higher-dose corticosteroids versus usual care, sotrovimab versus usual care, molnupiravir versus usual care, and nirmatrelvir–ritonavir versus usual care (appendix 5 pp 41–42). Participants and local study staff were not masked to the allocated treatment. Other than members of the Data Monitoring Committee, all individuals involved in the trial were masked to aggregated outcome data while recruitment and 28-day follow-up were ongoing.

### Procedures

Patients received usual standard of care plus empagliflozin 10 mg (Boehringer Ingelheim, Germany) orally daily, or usual care only, for 28 days in total or until discharge, whichever occurred earlier. A single online follow-up form was completed when participants were discharged, had died, or at 28 days after randomisation, whichever occurred earliest (appendix 5 pp 47–55). Information was recorded on adherence to allocated study treatment, receipt of other COVID-19 treatments, duration of admission, receipt of respiratory or renal support, and vital status (including cause of death). In addition, in the UK, routine health-care and registry data were obtained including information on vital status (with date and cause of death), discharge from hospital, receipt of respiratory support, or renal replacement therapy. For sites outside the UK a further case report form (appendix 5 pp 56–57) collected vital status at day 28 (if not already reported on follow-up form).

### Outcomes

Outcomes were assessed at 28 days after randomisation, with further analyses specified at 6 months. The primary outcome was all-cause mortality at 28 days. Secondary outcomes were time to discharge from hospital, and, among patients not on invasive mechanical ventilation at randomisation, invasive mechanical ventilation (including extra-corporal membrane oxygenation) or death. Prespecified subsidiary clinical outcomes were use of non-invasive respiratory support, time to successful cessation of invasive mechanical ventilation (defined as cessation of invasive mechanical ventilation within, and survival to, 28 days), use of renal dialysis or haemofiltration, cause-specific mortality, bleeding events, thrombotic events, major cardiac arrhythmias, thrombotic and bleeding events, other infections and metabolic complications (including ketoacidosis). Information on suspected serious adverse reactions was collected in an expedited fashion to comply with regulatory requirements.

### Statistical analysis

The intention for this comparison was to continue recruitment until sufficient primary outcomes had accrued to have 90% power to detect a proportional risk reduction of 20% at a two-sided significance level of 0·01. The steering committee reviewed the overall primary outcome rate (ie, in both arms combined to maintain blinding) and determined that at least 7000 patients would be required to provide such statistical power, allowing for non-adherence.

The independent data monitoring committee reviewed unblinded analyses of the study data and any other information considered relevant to the trial at intervals of around 2–3 months (depending on speed of enrolment) and was charged with determining if, in their view, the randomised comparisons in the study provided evidence on mortality that was strong enough (with a range of uncertainty around the results that was narrow enough) to affect national and global treatment strategies (appendix 5 p 58).

On March 3, 2023, the data monitoring committee recommended that the investigators review the unblinded data from the empagliflozin comparison (appendix 5 p 59). Consequently, recruitment to the empagliflozin comparison was closed on March 7, 2023.

The primary analysis for all outcomes was by intention-to-treat comparing patients randomly assigned to empagliflozin with patients randomised to usual care but for whom empagliflozin was both available and suitable as a treatment. For the primary outcome of 28-day mortality, the HR from an age-adjusted and respiratory status-adjusted Cox model was used to estimate the mortality rate ratio. We constructed Kaplan-Meier survival curves to display cumulative mortality over the 28-day period (starting on the day of randomisation and ending 28 days later). We used the same Cox regression method to analyse time to hospital discharge and successful cessation of invasive mechanical ventilation, with patients who died in hospital right-censored on day 29. Assessment of the proportional hazards assumption found no evidence against proportionality for any of the time to event outcomes. Median time to discharge was derived from Kaplan-Meier estimates. For the prespecified composite secondary outcome of progression to invasive mechanical ventilation or death within 28 days (among those not receiving invasive mechanical ventilation at randomisation), and the subsidiary clinical outcomes of receipt of ventilation and use of haemodialysis or haemofiltration, the precise dates were not available and so a log-binomial regression model was used to estimate the risk ratio (RR) adjusted for age and respiratory status. For safety outcomes, absolute risk differences were calculated as the difference in the proportions of patients experiencing outcomes by treatment allocation.

Prespecified subgroup analyses were performed for the primary outcome using the statistical test of interaction (test for heterogeneity or trend for three or more ordered groups), in accordance with the prespecified analysis plan, defined by the following characteristics at randomisation: age, sex, ethnicity, level of respiratory support, days since symptom onset, and use of corticosteroids (appendix 5 p 139). A sensitivity analysis restricted to patients with proven SARS-CoV-2 infection was also conducted.

Estimates of rate and RRs are shown with 95% CIs. All p values are two-sided and are shown without adjustment for multiple testing. The full database is held by the study team which collected the data from study sites and performed the analyses at the Nuffield Department of Population Health, University of Oxford (Oxford, UK).

Analyses were performed using SAS version 9.4 and R version 3.4. The trial is registered with ISRCTN (50189673) and ClinicalTrials.gov (NCT04381936).

### Role of the funding source

Neither the funders of the study nor Boehringer Ingelheim, which supplied empagliflozin for sites outside the UK, had any role in the study design, data collection, data analysis, data interpretation, or writing of the report.

## Results

**Figure 1:**
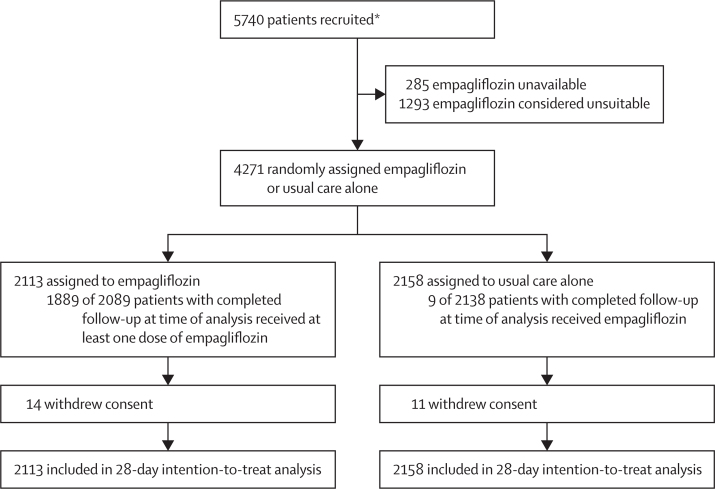
Trial profile *Number recruited overall during the period that adult participants could be recruited into empagliflozin comparison. Of the 4271 randomised to empagliflozin versus usual care, 1145 were additionally randomised to baricitinib versus usual care (554 [26%] of the empagliflozin group *vs* 591 [27%] of the usual care group); 321 were additionally randomised to higher-dose corticosteroids versus usual care (160 [8%] of the empagliflozin group *vs* 161 [7%] of the usual care group); 379 were additionally randomised to sotrovimab versus usual care (185 [9%] of the empagliflozin group *vs* 194 [9%] of the usual care group); 276 were additionally randomised to molnupiravir versus usual care (143 [7%] of the empagliflozin group *vs* 133 [6%] of the usual care group); and 47 patients were additionally randomised to nirmatrelvir–ritonavir versus usual care (19 [1%] of the empagliflozin group *vs* 28 [1%] of the usual care group). Of patients with completed follow-up at time of analysis, 1889 (90%) of 2089 patients in the empagliflozin group, and 9 (<1%) of 2138 patients in the usual care group, received empagliflozin.

Between July 28, 2021 and March 6, 2023, 4271 (74%) of 5740 patients enrolled into the RECOVERY trial were eligible to be randomly allocated to empagliflozin (ie, empagliflozin was available in the hospital at the time and the attending clinician was of the opinion that the patient had no known indication for, or contraindication to, empagliflozin; figure 1). The characteristics of the 1469 patients enrolled into the RECOVERY trial during this period but not included in the empagliflozin comparison are shown in appendix 5 pp 61–62. 2113 patients were randomly allocated to empagliflozin and 2158 were randomly allocated to usual care. The mean age of study participants in this comparison was 61·5 years (SD 16·4) and the median time since symptom onset was 8 days (IQR 5–11). At randomisation, 3842 (90%) of 4271 patients were receiving corticosteroids, approximately a quarter were receiving remdesivir and approximately a quarter had received tocilizumab (table 1).

**Table 1:** Baseline characteristics

		**Empagliflozin (n=2113)**	**Usual care (n=2158)**
Age, years		61·1 (16·3)	61·8 (16·4)
	<70	1412 (67%)	1393 (65%)
	≥70 to <80	434 (21%)	479 (22%)
	≥80	267 (13%)	286 (13%)
Sex			
	Male	1326 (63%)	1339 (62%)
	Female	781 (37%)	817 (38%)
	Not recorded	6 (<1%)	2 (<1%)
Country			
	Ghana	2 (<1%)	2 (<1%)
	India	24 (1%)	19 (1%)
	Indonesia	68 (3%)	68 (3%)
	Nepal	139 (7%)	119 (6%)
	South Africa	10 (<1%)	14 (1%)
	Viet Nam	40 (2%)	53 (2%)
	UK	1830 (87%)	1883 (87%)
Ethnicity			
	White	1557 (74%)	1607 (74%)
	Black, Asian, and minority ethnic	361 (17%)	330 (15%)
	Unknown	195 (9%)	221 (10%)
Number of days since symptom onset		8 (5–11)	8 (5–12)
Number of days since hospitalisation		2 (1–3)	2 (1–3)
Respiratory support received			
	None	255 (12%)	260 (12%)
	Simple oxygen	1317 (62%)	1383 (64%)
	Non invasive ventilation	512 (24%)	500 (23%)
	Invasive mechanical ventilation	29 (1%)	15 (1%)
Biochemistry			
	C-reactive protein, mg/L	83 (39–148)	85 (38–151)
	Creatinine, μmol/L	75 (62–94)	78 (63–96)
Previous diseases			
	Diabetes	333 (16%)	356 (16%)
	Heart disease	471 (22%)	455 (21%)
	Chronic lung disease	533 (25%)	508 (24%)
	Tuberculosis	9 (<1%)	7 (<1%)
	HIV	21 (1%)	13 (1%)
	Severe liver disease*	20 (1%)	21 (1%)
	Severe kidney impairment†	66 (3%)	80 (4%)
	Any of the above	1014 (48%)	1039 (48%)
SARS-CoV-2 PCR test result			
	Positive	2046 (97%)	2097 (97%)
	Negative	12 (1%)	10 (<1%)
	Unknown	55 (3%)	51 (2%)
Received a COVID-19 vaccine		1412 (67%)	1453 (67%)
Use of other treatments			
	Corticosteroids	1910 (90%)	1932 (90%)
	Remdesivir	541 (26%)	547 (25%)
	Tocilizumab	504 (24%)	491 (23%)
	Plan to use tocilizumab within the next 24 h	208 (10%)	240 (11%)
Other randomly assigned treatments			
	Baricitinib	554 (26%)	591 (27%)
	High dose steroids	160 (8%)	161 (7%)
	Sotrovimab	185 (9%)	194 (9%)
	Molnupiravir	143 (7%)	133 (6%)
	Nirmatrelvir–ritonavir	19 (1%)	28 (1%)

Results are n (%), mean (SD), or median (IQR).

* Defined as requiring ongoing specialist care.

† Defined as estimated glomerular filtration rate <30 mL/min per 1·73 m^2^.

The follow-up form was completed for 2089 (99%) of 2113 patients in the empagliflozin group and 2138 (99%) of 2158 patients in the usual care group. Among patients with a completed follow-up form, 1889 (90%) of 2089 allocated to empagliflozin received at least one dose and, of these, 1321 (70%) received it on most (≥90%) days of their admission (or until 28 days after randomisation if not discharged sooner; figure 1; appendix 5 p 63). By comparison, less than 1% of those allocated to usual care alone received any dose of empagliflozin. Use of other treatments for COVID-19 was similar among patients allocated empagliflozin and among those allocated usual care (appendix 5 p 63).

**Figure 2:**
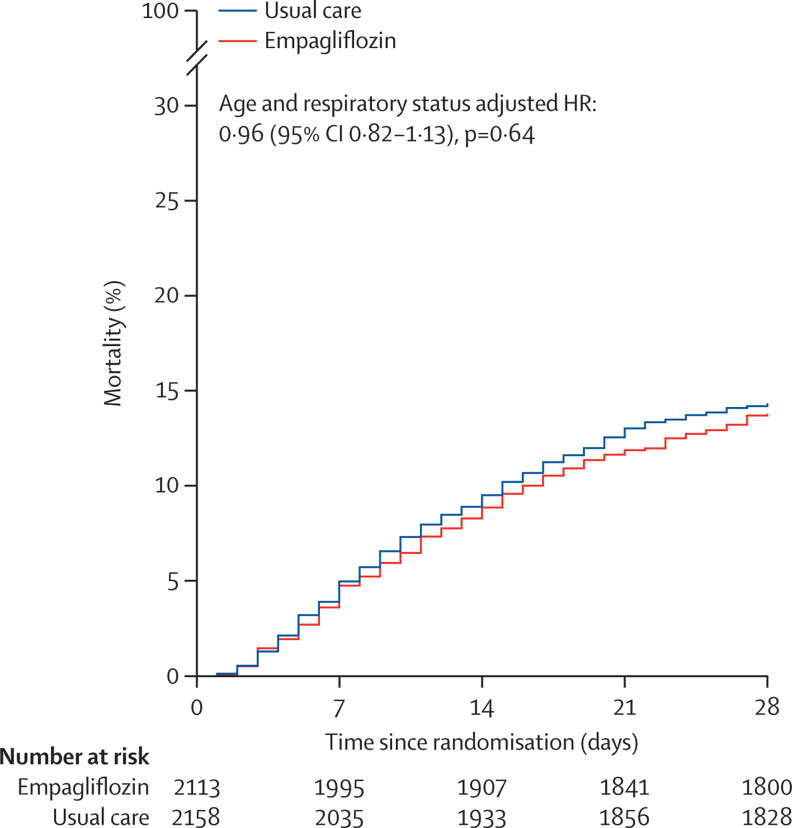
Effect of allocation to empagliflozin on 28-day mortality HR=hazard ratio.

**Figure 3:**
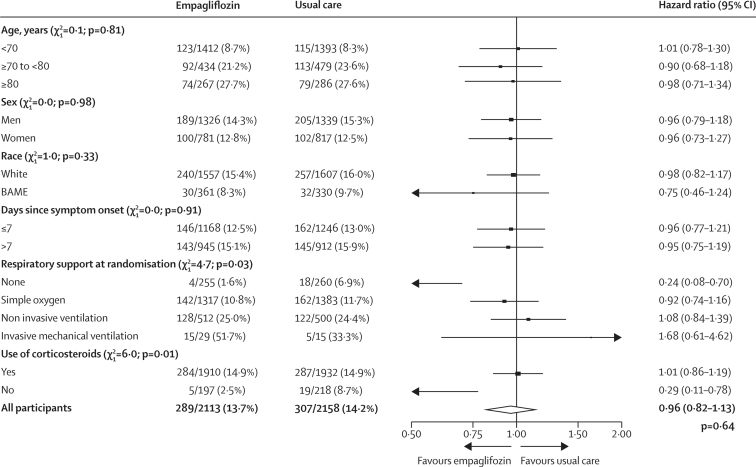
Effect of allocation to empagliflozin on 28-day mortality by baseline characteristics Subgroup-specific rate ratio estimates are represented by squares (with areas of the squares proportional to the amount of statistical information) and the lines through them correspond to the 95% CIs. The ethnicity, days since onset, and use of corticosteroids subgroups exclude those with missing data, but these patients are included in the overall summary diamond. BAME=Black, Asian, and minority ethnic.

Primary and secondary outcome data were known for greater than 99% of randomly assigned patients. There was no evidence of a significant difference in the proportion of patients who met the primary outcome of 28-day mortality between the two randomised groups (289 [14%] of 2113 patients in the empagliflozin group *vs* 307 [14%] of 2158 patients in the usual care group; HR 0·96 [95% CI 0·82–1·13]; p=0·64; figure 2). We observed similar results across all prespecified subgroups (figure 3), except among the small group of patients not requiring oxygen at baseline or not receiving corticosteroids among whom there were very few (approximately 20) events. A post hoc subgroup analysis also found no evidence that the effect of empagliflozin varied in patients with diabetes and without diabetes (HR 1·23 [95% CI 0·86–1·77] for patients with diabetes and 0·91 [0·76–1·09] for patients without diabetes; p_heterogeneity_=0·15). The result of a sensitivity analysis restricted to patients with laboratory-confirmed SARS-CoV-2 infection (HR 0·97 [0·82–1·14]) was not materially different to the overall result.

The median time to discharge from hospital alive was 8 days (IQR 5–19) in both groups and there was no difference in the probability of being discharged alive within 28 days (79% in the empagliflozin group *vs* 78% in the usual care group, rate ratio 1·03, [95% CI 0·96–1·10]; p=0·44; table 2). Among those not on invasive mechanical ventilation at baseline, the number of patients progressing to the prespecified composite secondary outcome of invasive mechanical ventilation or death was similar in both groups (338 [16%] *v*s 371 [17%], RR 0·95 [95% CI 0·84–1·08]; p=0·44). Similar results were seen in all prespecified subgroups of patients (appendix 5 pp 66–67).

**Table 2:** Effect of allocation to empagliflozin on key study outcomes

		**Empagliflozin (n=2113)**	**Usual care (n=2158)**	**Effect size (95% CI)**	**p value**
**Primary outcome**					
28-day mortality		289 (13·7%)	307 (14·2%)	HR 0·96 (0·82–1·13)	0·64
**Secondary outcomes**					
Median time to being discharged alive, days		8 (5–19)	8 (5–20)	..	..
Discharged from hospital within 28 days		1678 (79·4%)	1677 (77·7%)	HR 1·03 (0·96–1·10)	0·44
Receipt of invasive mechanical ventilation or death*		338/2084 (16·2%)	371/2143 (17·3%)	RR 0·95 (0·84–1·08)	0·44
	Invasive mechanical ventilation	130/2084 (6·2%)	133/2143 (6·2%)	RR 0·97 (0·77–1·21)	0·77
	Death	274/2084 (13·1%)	302/2143 (14·1%)	RR 0·96 (0·84–1·11)	0·59
**Subsidiary clinical outcomes**					
Receipt of ventilation†		245/1567 (15·6%)	259/1641 (15·8%)	RR 1·00 (0·85–1·17)	0·97
	Non-invasive ventilation	237/1572 (15·0%)	252/1643 (15·3%)	RR 0·99 (0·84–1·16)	0·90
	Invasive mechanical ventilation	51/1567 (3·3%)	49/1641 (3·0%)	RR 1·09 (0·74–1·60)	0·66
Successful cessation of invasive mechanical ventilation‡		10/29 (34·5%)	6/15 (40·0%)	HR 0·67 (0·24–1·85)	0·44
Renal replacement therapy§		45/2103 (2·1%)	44/2146 (2·1%)	RR 0·96 (0·64–1·45)	0·86

Data are n (%) or median (IQR). HR=hazard ratio. RR= risk ratio.

* Analyses exclude those on invasive mechanical ventilation at randomisation.

† Analyses exclude those on any form of ventilation at randomisation.

‡ Analyses restricted to those on invasive mechanical ventilation at randomisation.

§ Analyses exclude those on haemodialysis or haemofiltration at randomisation.

We found no evidence of differences in the prespecified subsidiary clinical outcomes of cause-specific mortality (appendix 5 p 64), use of ventilation, or successful cessation of invasive mechanical ventilation between treatment groups (table 2). We found no evidence of a difference in the incidence of acute kidney injury (defined as an increase in the pre-randomisation creatinine concentration of ≥50%; table 3), or need for renal dialysis or haemofiltration between treatment groups (table 2). The incidence of new cardiac arrhythmias, bleeding events, and non-coronavirus infections was also similar in the two groups (table 3). There were fewer thrombotic events among patients allocated to empagliflozin than usual care (52 [2·5%] of 2113 for empagliflozin *vs* 84 [3·9%] of 2158 for usual care, absolute difference –1·4% [–2·5 to –0·4]; table 3). The incidence of metabolic complications were similar in the two groups, with reported ketoacidosis in five (0·2%) *vs* two (0·1%) patients. There were two reports of a serious adverse reaction believed to be related to empagliflozin, both were ketosis without acidosis (including one patient without diabetes) and resolved rapidly on cessation of the drug.

**Table 3:** Effect of allocation to empagliflozin on new cardiac arrhythmia, thrombotic events, clinically significant bleeds, non-coronavirus infections, and metabolic complications

		**Empagliflozin (n=2113)**	**Usual care (n=2158)**	**Absolute percent difference (95% CI)**
Number with follow-up form		2089	2138	..
New cardiac arrhythmia				
	Atrial flutter or fibrillation	46 (2·2%)	39 (1·8%)	−0·4 (−0·5 to 1·2)
	Other supraventricular tachycardia	4 (0·2%)	9 (0·4%)	−0·2 (−0·6 to 0·1)
	Subtotal: supraventricular tachycardia	49 (2·3%)	48 (2·2%)	−0·1 (−0·8 to 1·0)
	Ventricular tachycardia	5 (0·2%)	2 (0·1%)	0·1 (−0·1 to 0·4)
	Ventricular fibrillation	0	1 (0·0%)	−0·0 (−0·1 to 0·0)
	Subtotal: ventricular tachycardia or fibrillation	5 (0·2%)	3 (0·1%)	0·1 (−0·2 to 0·4)
	Atrioventricular block requiring intervention	1 (0·0%)	1 (0·0%)	0·0 (−0·1 to 0·1)
	Total: any major cardiac arrhythmia	54 (2·6%)	53 (2·5%)	0·1 (−0·8 to 1·0)
Thrombotic events				
	Pulmonary embolism	47 (2·2%)	64 (3·0%)	−0·7 (−1·7 to 0·2)
	Deep-vein thrombosis	4 (0·2%)	11 (0·5%)	−0·3 (−0·7 to 0·0)
	Ischaemic stroke	2 (0·1%)	7 (0·3%)	−0·2 (−0·5 to 0·0)
	Myocardial infarction	0	4 (0·2%)	−0·2 (−0·4 to −0·0)
	Systemic arterial embolism	0	1 (0·0%)	−0·0 (−0·1 to 0·0)
	Subtotal: any thrombotic event	52 (2·5%)	84 (3·9%)	−1·4 (−2·5 to −0·4)
Clinically significant bleeds				
	Intra-cranial	1 (0·0%)	3 (0·1%)	−0·1 (−0·3 to 0·1)
	Gastrointestinal	7 (0·3%)	10 (0·5%)	−0·1 (−0·5 to 0·2)
	Other or unrecorded site	11 (0·5%)	8 (0·4%)	0·1 (−0·3 to 0·5)
	Requiring blood transfusion	15 (0·7%)	18 (0·8%)	−0·1 (−0·6 to 0·4)
	Requiring surgery	1 (0·0%)	0	0·0 (−0·0 to 0·1)
	Requiring endoscopy	5 (0·2%)	1 (0·0%)	0·2 (−0·0 to 0·4)
	Requiring vasoactive drugs	4 (0·2%)	1 (0·0%)	0·1 (−0·1 to 0·3)
	Subtotal: any clinically significant bleeding	18 (0·9%)	21 (1·0%)	−0·1 (−0·7 to 0·4)
Non-coronavirus infection				
	Pneumonia	144 (6·8%)	143 (6·6%)	0·2 (−1·3 to 1·7)
	Urinary tract	31 (1·5%)	31 (1·4%)	0·0 (−0·7 to 0·7)
	Biliary	0	3 (0·1%)	−0·1 (−0·3 to 0·0)
	Other intra-abdominal	2 (0·1%)	4 (0·2%)	−0·1 (−0·3 to 0·1)
	Blood stream	25 (1·2%)	25 (1·2%)	0·0 (−0·6 to 0·7)
	Skin	9 (0·4%)	14 (0·6%)	−0·2 (−0·7 to 0·2)
	Other	48 (2·3%)	46 (2·1%)	0·1 (−0·7 to 1·0)
	Subtotal: any non-coronavirus infection	54 (2·6%)	53 (2·5%)	0·1 (−0·8 to 1·0)
Metabolic complications				
	Ketoacidosis	5 (0·2%)	2 (0·1%)	0·1 (−0·1 to 0·4)
	Hyperglycaemic hyperosmolar state	9 (0·4%)	14 (0·6%)	−0·2 (−0·7 to 0·2)
	Other hyperglycaemia requiring new use of insulin	123 (5·8%)	132 (6·1%)	−0·3 (−1·7 to 1·1)
	Severe hypoglycaemia	4 (0·2%)	7 (0·3%)	−0·1 (−0·4 to 0·2)
Acute kidney injury*				
	Stage 1	44/2103 (2·1%)	35/2146 (1·6%)	0·5 (−0·4 to 1·3)
	Stage 2	16/2103 (0·8%)	23/2146 (1·1%)	−0·3 (−0·9 to 0·3)
	Stage 3	72/2103 (3·4%)	72/2146 (3·4%)	0·1 (−1·0 to 1·2)
	Subtotal: any acute kidney injury	132/2103 (6·3%)	130/2146 (6·1%)	0·2 (−1·2 to 1·7)

Data are n (%).

* Analyses exclude those on haemodialysis or haemofiltration at randomisation.

## Discussion

In this large randomised trial involving over 4000 patients from seven countries and nearly 600 deaths, allocation to empagliflozin was not associated with reductions in mortality, duration of hospitalisation, or the risk of being ventilated or dying for those not on ventilation at baseline. These results were consistent across prespecified subgroups of age, sex, race, and duration of symptoms before randomisation.

The benefits of immunomodulatory therapies in patients with moderate to severe COVID-19 demonstrates the importance of inflammation in this patient group and empagliflozin was proposed as a treatment for COVID-19 partly based on its anti-inflammatory activity as well as purported benefits on endothelial function and cellular energy metabolism.^28^ The lack of evidence of benefit from empagliflozin in this large well-powered trial suggests that these properties of empagliflozin are either insufficient to produce a meaningful reduction in mortality risk or are not affecting the relevant pathways in moderate to severe COVID-19. These putative mechanisms were first demonstrated in patients with diabetes who only constituted 16% of the RECOVERY trial population, so the overall result observed in RECOVERY might have been due to the benefit from these mechanisms being restricted to patients with diabetes. However, there was no evidence of different effects on the primary outcome in patients with and without diabetes in a post hoc subgroup analysis, and the known benefits of SGLT2 inhibitors in patients with heart failure and chronic kidney disease are also not modified by the presence or absence of diabetes.^2^ These results are in contrast to non-randomised studies which suggested that use of SGLT2 inhibitors before infection with SARS-CoV-2 was associated with a reduced risk of death.^29^ This difference might be because the timing of treatment initiation is important, or because the positive findings from non-randomised studies are the result of biases inherent in such study designs.^30^ There is weak evidence that there might be some benefit for 28-day mortality in patients not receiving a corticosteroid or not requiring oxygen, which are mostly the same patients. However, this observation is based on a very small number of events, marginally significant tests for heterogeneity or trend, and is not supported by either of the secondary outcomes.

To our knowledge, only one other trial of an SGLT2 inhibitor in COVID-19 has been reported to date. The DARE-19 trial recruited 1250 patients hospitalised (but not critically ill) with COVID-19 and with at least one cardiometabolic risk factor (ie, hypertension, type 2 diabetes, atherosclerotic cardiovascular disease, heart failure, or chronic kidney disease).^17^ There was no significant effect of dapagliflozin on either of the two dual primary outcomes (new or worsened organ function or death, and change in clinical status by day 30), although there were numerically fewer poor outcomes in the dapagliflozin group, including 41 deaths versus 54 in the placebo group. However, DARE-19 was not large enough to detect plausibly moderate benefits of treatment.

Trials of SGLT2 inhibitors in a chronic disease setting have found consistent evidence of a reduction in acute kidney injury. A meta-analysis of 13 large placebo-controlled trials including over 2000 acute kidney injury events reported a reduction of nearly one-quarter (relative risk 0·77 [95% CI 0·70–0·84]) in this outcome.^2^ The RECOVERY trial did not find evidence of benefit (or harm) of empagliflozin on the risk of developing acute kidney injury in the acute setting where the injury could have already begun before randomisation. This result is consistent with that of DARE-19 which also found no evidence of a benefit (or harm) on acute kidney injury (HR 0·65 [95% CI 0·50–1·07]).^31^

Strengths of this trial include that it was randomised, had a large sample size, broad eligibility criteria, was international, and more than 99% of patients were followed up for the primary outcome. However, detailed information on laboratory markers of inflammation and immune response was not collected, nor was information on radiological or physiological outcomes. Although this randomised trial is open label (ie, participants and local hospital staff are aware of the assigned treatment), the outcomes are unambiguous and were ascertained without bias through linkage to routine health records in the large majority of patients.

The RECOVERY trial only studied patients who had been hospitalised with COVID-19 and, therefore, is not able to provide any evidence on the safety and efficacy of empagliflozin used in other patient groups. Due to the recommendation that empagliflozin be taken orally (and not via a gastric feeding tube), there were few patients recruited requiring invasive mechanical ventilation. Nevertheless, the reassuring safety findings in RECOVERY suggest that empagliflozin can be safely used in the acute setting and do not need to be routinely discontinued if there is an appropriate indication. These results show that the key risk of ketoacidosis can be safely mitigated with simple monitoring and advice to managing physicians.

In summary, the results of this large, randomised trial do not support the use of empagliflozin as a treatment for adults hospitalised with COVID-19.

**This online publication has been corrected. The corrected version first appeared at thelancet.com/diabetes-endocrinology on November 28, 2023**

## Data sharing

The protocol, consent form, statistical analysis plan, definition and derivation of clinical characteristics and outcomes, training materials, regulatory documents, and other relevant study materials are available online at http://www.recoverytrial.net. As described in the protocol, the Trial Steering Committee will facilitate the use of the study data and approval will not be unreasonably withheld. Deidentified participant data will be made available to bona fide researchers registered with an appropriate institution within 3 months of publication. However, the Steering Committee will need to be satisfied that any proposed publication is of high quality, honours the commitments made to the study participants in the consent documentation and ethical approvals, and is compliant with relevant legal and regulatory requirements (eg, relating to data protection and privacy). The Steering Committee will have the right to review and comment on any draft manuscripts before publication. Data will be made available in line with the policy and procedures described at https://www.ndph.ox.ac.uk/data-access. Those wishing to request access should complete the form at https://www.ndph.ox.ac.uk/files/about/data_access_enquiry_form_13_6_2019.docx and e-mail to data.access@ndph.ox.ac.uk.

## Declaration of interests

NS, RH and MJL are named on grants to the University of Oxford from Boehringer Ingelheim for other research projects. All other authors declare no competing interests.

## Supplementary Materials

Nepali translation of the abstract

Hindi translation of the abstract

Indonesian (Bahasa) translation of the abstract

Vietnamese translation of the abstract
