# Correction to *Lancet Diabetes Endocrinol* 2023; 11: 905–14

**DOI:** 10.1016/S2213-8587(23)00360-1

**Published:** 2024-01

*RECOVERY Collaborative Group. Empagliflozin in patients admitted to hospital with COVID-19 (RECOVERY): a randomised, controlled, open-label, platform trial.* Lancet Diabetes Endocrinol *2023;* 11: *905–14*—In the Summary of this Article, the Funding line should have read “UK Research and Innovation (Medical Research Council) and National Institute of Health Research (MC_PC_19056), Wellcome Trust (222406/Z/20/Z), and Foreign, Commonwealth and Development Office (Project Number 204765-112)”. In the Acknowledgments, the funding information for the Foreign, Commonwealth and Development office has been corrected. These corrections has been made to the online version as of Nov 28, 2023.

