# Supplementary material for: Empagliflozin in patients admitted to hospital with COVID-19 (RECOVERY): a randomised, controlled, open-label, platform trial
Source: Lancet Diabetes Endocrinol. 2023 Dec;11(12):905–14. doi: 10.1016/S2213-8587(23)00253-X (PMC10957483; doi:10.1016/S2213-8587(23)00253-X)
Supplement: Nepali translation of the abstract [file mmc1.pdf]

THE LANCET
Diabetes & Endocrinology

Supplementary appendix 1

This translation in Nepali was submitted by the authors and we reproduce it as supplied. It has not been peer reviewed. *The Lancet* editorial processes have only been applied to the original in English, which should serve as reference for this manuscript.

यो अनुवाद नेपाली भाषामा लेखकहरूद्वारा बुझाइएको हो र हामीले हुबहु उपलब्ध गराएका छौं। यो अनुवादलाई सहकर्मी समीक्षा (peer reviewed) गरिएको छैन। ल्यान्सेट (Lancet) को सम्पादकीय प्रक्रियाहरू मूल अंग्रेजी भाषाको अनुवादमा मात्र लागू गरिएको छ, जसले यस पाण्डुलिपि (manuscript) को लागि सन्दर्भको रूपमा काम गर्छ।

Supplement to: RECOVERY Collaborative Group. Empagliflozin in patients admitted to hospital with COVID-19 (RECOVERY): a randomised, controlled, open-label, platform trial. *Lancet Diabetes Endocrinol* 2023; published online Oct 18. https://doi.org/10.1016/S2213-8587(23)00253-X.

कोभिड १९ का कारण अस्पतालमा भर्ना भएका बिरामीहरूमा एम्पाग्लिफ्लोजिन (RECOVERY): रान्डमाइज्ड, कन्ट्रोल्ड, ओपन लेबल, प्लेटफर्म ट्रायल

RECOVERY कोलाबोरेटिभ समूह*

सारांश

पृष्ठभूमि : एम्पाग्लिफ्लोजिन (Empagliflozin) को एन्टी-इन्फ्लामेटरी, मेटाबोलिक र हेमोडाईनामिक (anti-inflammatory, metabolic and haemodynamic) प्रभावहरु भएको कारणले गर्दा यसलाई कोभिड १९ को उपचारको लागि प्रस्ताव गरिएको हो। रिकभरी ट्रायल (RECOVERY trial) को उद्देश्य कोभिड १९ का कारण अस्पतालमा भर्ना भएका बिरामीहरूमा एम्पाग्लिफ्लोजिनको सुरक्षा र प्रभावकारिताको मूल्याङ्कन गर्नु हो।

विधिहरू: यस रान्डमाइज्ड, कन्ट्रोल्ड, ओपन लेबल (randomised, controlled, open label)) रिकभरी ट्रायलमा कोभिड १९ का कारण अस्पतालमा भर्ना भएका बिरामीहरूमा कोभिड १९ विरुद्धका विभिन्न सम्भावित उपचारहरुलाई सामान्यता दिइने उपचार (usual care) सँग तुलना गरिएको थियो। यस विश्लेषणमा, हामीले भाग लिन योग्य र सहमत हुनुभएका वयस्कहरूलाई (adults) वेबमा आधारित (web based) सरल (अस्तरीकृत) रान्डमाइजेसन (randomization) प्रयोग गरी आवंटन (allocation) को बारेमा थाहा नदिई ट्रायलमा अनियमित रूपमा (randomly) १:१ अनुपातमा, या सामान्यता दिइने उपचार वा सामान्यता दिइने उपचारसँगै दैनिक एक चोटी मुखबाट १० mg एम्पाग्लिफ्लोजिन २८ दिनसम्म वा अस्पतालबाट डिस्चार्ज नभएसम्म (जुन पहिले आयो तेहि) को लागि छुट्टाइएको थियो। यस ट्रायलको प्राथमिक परिणाम २८-दिन मृत्युदर र माध्यमिक परिणामहरू अस्पताल भर्नाको अवधि र (प्रारम्भिक चरणमा इनभेसिभ मेकानिकल भेन्टिलेसन (invasive mechanical ventilation) मा नभएका सहभागीहरू बीच) इनभेसिभ मेकानिकल भेन्टिलेशन वा मृत्युको समग्र थियो । मार्च ३ २०२३ मा, स्वतन्त्र डाटा रेख देख गर्ने समुह (data monitoring committee) ले अनुसन्धानकर्ताहरूलाई तथ्याङ्कको समीक्षा गर्न सिफारिस गरेको थियो र फलस्वरूप ७ मार्च २०२३मा ट्रायलमा सहभागिता रोकेको थियो । यो चलिरहेको रिकभरी ट्रायल ISRCTN (50189673) र ClinicalTrials.gov (NCT04381936) मा दर्ता गरिएको छ।

नतिजा : जुलाई २८, २०२१ देखि मार्च ६, २०२३ सम्ममा, ४२७१ बिरामीहरू एम्पाग्लिफ्लोजिन (२११३ बिरामीहरू) वा सामान्यता दिइने उपचार (२१५८ बिरामीहरू) पाउनको लागि अनियमित रूपमा आवंटित भएका थिए । ९९ % भन्दा बढी अनियमित रूपमा आवंटन गरीएको बिरामीहरूको प्राथमिक र माध्यमिक परिणामको डाटाको बारेमा थाहा थियो । समग्रमा, २११३ बिरामीहरू मध्य एम्पाग्लिफ्लोजिन पाउने २८९ (१४%) बिरामीहरू र २१५८ बिरामीहरू मध्य सामान्यता दिइने उपचार पाउने ३०७ (१४%) बिरामीहरूको २८ दिनभित्र मृत्यु भएको थियो (rate ratio 0.९६ [९५% CI 0.८२ - १. १३]; p= 0. ६४) । अस्पताल भर्नाको अवधि (दुबै समूहको लागि median ८ दिन) वा २८ दिनभित्र अस्पतालबाट डिस्चार्ज हुने बिरामीको अनुपात (१६७८ [७९%] एम्पाग्लिफ्लोजिन पाउने समूह vs. १६७७ [७८ %] सामान्यता दिइने उपचार पाउने समूह; rate ratio १.0३ [९५% CI 0.९६-१.१0]; p=0. ४४) मा महत्त्वपूर्ण सांख्यिकीय भिन्नता थिएन। प्रारम्भिक चरण (baseline) मा इनभेसिभ मेकानिकल भेन्टिलेशनमा नभएकाहरूमा, इनभेसिभ मेकानिकल भेन्टिलेशन वा मृत्युको समग्र अन्तिम बिन्दु (composite endpoint) को अनुपातमा महत्त्वपूर्ण सांख्यिकीय भिन्नताको कुनै प्रमाण थिएन (२०८४ मध्य ३३८ [१६%] vs. २१४३ मध्य ३७१ [१७%]; risk ratio 0. ९५ [९५% CI 0. ८४ - १.0८]; p=0. ४४)। एम्पाग्लिफ्लोजिनसँग सम्बन्धित दुईवटा गम्भीर प्रतिकूल घटनाहरू (serious adverse events) रिपोर्ट गरिएको थियो: दुवै एसिडोसिस बिना केटोसिस (ketosis without acidosis) थिए।

व्याख्या: कोभिड १९ का कारण अस्पतालमा भर्ना भएका वस्यकहरूमा, एम्पाग्लिफ्लोजिनको प्रयोगको २८ दिन मृत्यु दर, अस्पताल भर्नाको अवधि वा इनभेसिभ मेकानिकल भेन्टिलेशनमा जाने जोखिम वा मृत्युको जोखिम घटाउनेसँग सम्बन्ध नभएकोले, यस्ता बिरामीहरूमा यो उपचार प्रयोग नगर्न संकेत दिइएको छ जब सम्म मधुमेह जस्ता अरु अवस्थाहरूको लागि कुनै आधार स्थापित हुदैन ।

कोष: यु के रिसर्च एण्ड इनोभेशन (मेडिकल रिसर्च काउन्सिल) र नेशनल इन्स्टिच्युट अफ हेल्थ रिसर्च (MC_PC_19056), र वेल्कम ट्रस्ट (:222406/Z/20/Z) (UK Research and Innovation (Medical Research Council) and National Institute of Health Research (Grant ref: MC_PC_19056), and Wellcome Trust (Grant Ref: 222406/Z/20/Z))
