# Supplementary material for: Empagliflozin in patients admitted to hospital with COVID-19 (RECOVERY): a randomised, controlled, open-label, platform trial
Source: Lancet Diabetes Endocrinol. 2023 Dec;11(12):905–14. doi: 10.1016/S2213-8587(23)00253-X (PMC10957483; doi:10.1016/S2213-8587(23)00253-X)
Supplement: Hindi translation of the abstract [file mmc2.pdf]

THE LANCET
Diabetes & Endocrinology

Supplementary appendix 2

This translation in Hindi was submitted by the authors and we reproduce it as supplied. It has not been peer reviewed. *The Lancet*'s editorial processes have only been applied to the original in English, which should serve as reference for this manuscript.

हिंदी में यह अनुवाद लेखकों द्वारा प्रस्तुत किया गया था और हम इसे जैसे उपलब्ध कराया गया वैसे पुन: पेश करते हैं। इस पर सहकर्मी की समीक्षा नहीं की गई है। लैंसेट की संपादकीय प्रक्रियाओं को केवल अंग्रेजी में मूल पर लागू किया गया है, जो इस पांडुलिपि के संदर्भ के रूप में काम आना चाहिए।

Supplement to: RECOVERY Collaborative Group. Empagliflozin in patients admitted to hospital with COVID-19 (RECOVERY): a randomised, controlled, open-label, platform trial. *Lancet Diabetes Endocrinol* 2023; published online Oct 18. https://doi.org/10.1016/S2213-8587(23)00253-X.

अस्पताल में भर्ती कोविड-19 के रोगियों में एम्पाग्लिफ्लोज़िन (रिकवरी): एक रैन्डमाइज़्ड, नियंत्रित, ओपन-लेबल, प्लेटफ़ॉर्म परीक्षण

रिकवरी सहयोगात्मक समूह*

सारांश

परिप्रेक्ष्य: एम्पाग्लिफ्लोज़िन को इसके एंटी-इंफ्लेमेटरी, मेटाबोलिक, और हेमोडायनामिक प्रभावों के आधार पर कोविड-19 के उपचार के रूप में प्रस्तावित किया गया है। रिकवरी परीक्षण का उद्देश्य अस्पताल में भर्ती कोविड-19 रोगियों में इसकी सुरक्षा और प्रभावकारिता का आकलन करना था।

पद्यतियां: इस रैन्डमाइज़्ड नियंत्रित, ओपन-लेबल रिकवरी परीक्षण में, अस्पताल में भर्ती कोविड-19 रोगियों में कई संभावित उपचारों की तुलना सामान्य देखभाल से की जाती है। इस विश्लेषण में योग्य और सहमति देने वाले ≥18 वर्ष के रोगियों को 1:1 के अनुपात में रैन्डमाइज़्ड रूप से, केवल सामान्य देखभाल या सामान्य देखभाल के साथ एम्पाग्लिफ्लोज़िन 10 मिलीग्राम मौखिक रूप से प्रतिदिन एक बार 28 दिनों के लिए या डिस्चार्ज होने तक (इनमें से जो भी पहले हो) वेब-आधारित सिंपल (अन्स्ट्रैटिफाइड) रैन्डमाइज़ेशन और एलोकेशन कन्सीलमेंट का उपयोग करते हुए दो समूह में बांटा गया था। प्राथमिक परिणाम 28 दिन की मृत्यु दर था; द्वितीयक परिणाम अस्पताल में भर्ती होने की अवधि और (बेसलाइन पर इनवेसिव मैकेनिकल वेंटिलेशन पर नहीं रहने वाले प्रतिभागियों के बीच) इनवेसिव मैकेनिकल वेंटिलेशन या मृत्यु का सम्मिश्रण थे। मार्च 3, 2023 को निष्पक्ष डेटा निगरानी समिति ने अनुशंसित किया कि जांचकर्ता डेटा की समीक्षा करें और परिणामस्वरूप मार्च 7, 2023 को भर्ती रोक दी गई। वर्तमान में जारी रिकवरी परीक्षण ISRCTN (50189673) और ClinicalTrials.gov (NCT04381936) के साथ पंजीकृत है।

परिणाम: जुलाई 28, 2021 से मार्च 6, 2023 के बीच, 4271 रोगियों को एम्पाग्लिफ्लोज़िन (2113 रोगी) या केवल सामान्य देखभाल (2158 रोगी) प्राप्त करने के लिए रैन्डमाइज़्ड रूप से बांटा गया था। प्राथमिक और द्वितीयक परिणाम डेटा 99% से अधिक रैंडम रूप से निर्दिष्ट रोगियों के लिए ज्ञात थे। कुल मिलाकर, एम्पाग्लिफ्लोज़िन लेने वाले 2113 रोगियों में से 289 (14%) और सामान्य देखभाल लेने वाले 2158 रोगियों में से 307 (14%) की 28 दिनों के भीतर की मृत्यु हो गई (रेट रेश्यो

0·96; [95% सीआई 0·82-1·13]; पी=0·64)। अस्पताल में भर्ती होने की अवधि (मीडियन 8 दिन दोनों समूहों के लिए) या 28 दिनों के भीतर अस्पताल से छुट्टी पाने वाले जीवित रोगियों के प्रोपोरशन (1678 [79%] एम्पाग्लिफ़्लोज़िन समूह के प्रति 1677 [78%] सामान्य देखभाल समूह में; रेट रेश्यो 1·03 [95% सीआई 0·96-1·10]; पी=0·44) में महत्वपूर्ण अंतर का कोई सबूत नहीं था । बेसलाइन पर इनवेसिव मैकेनिकल वेंटिलेशन पर नहीं रहने वालों रोगियों में, इनवेसिव मैकेनिकल वेंटिलेशन या मृत्यु के कम्पोज़िट एण्डपॉइंट तक पहुँचने वाले प्रोपोरशन (2084 में से 338 [16%] of 2084 के प्रति 2143 में से 371 [17%]; रिस्क रेश्यो 0·95; [95% सीआई 0·84-1·08]; पी=0·44) में महत्वपूर्ण अंतर का कोई सबूत नहीं था। एम्पाग्लिफ्लोज़िन से सम्भवतः संबंधित दो गंभीर प्रतिकूल घटनाएं बताई गई थीं: दोनों एसिडोसिस के बिना केटोसिस थे।

व्याख्या: कोविड-19 के साथ अस्पताल में भर्ती ≥18 वर्ष के रोगियों में, एम्पाग्लिफ्लोज़िन, 28 दिनों की मृत्यु दर में कमी, अस्पताल में रहने की अवधि, या इनवेसिव मैकेनिकल वेंटिलेशन की ओर बढ़ने के जोखिम या मृत्यु से संबंधित नहीं था; इसलिए जब तक कि मधुमेह जैसी किसी भिन्न स्थिति का कोई स्थापित संकेत न हो, ऐसे रोगियों के इलाज के लिए इसका संकेत नहीं दिया जाता है ।

अनुदान: यू.के. रिसर्च एंड इनोवेशन (मेडिकल रिसर्च काउंसिल) और नेशनल इंस्टीट्यूट ऑफ हेल्थ रिसर्च (एमसी_पीसी _19056), और वेलकम ट्रस्ट (222406/Z/20/Z)
