# Supplementary material for: Empagliflozin in patients admitted to hospital with COVID-19 (RECOVERY): a randomised, controlled, open-label, platform trial
Source: Lancet Diabetes Endocrinol. 2023 Dec;11(12):905–14. doi: 10.1016/S2213-8587(23)00253-X (PMC10957483; doi:10.1016/S2213-8587(23)00253-X)
Supplement: Indonesian (Bahasa) translation of the abstract [file mmc3.pdf]

THE LANCET
Diabetes & Endocrinology

Supplementary appendix 3

This translation in Bahasa was submitted by the authors and we reproduce it as supplied. It has not been peer reviewed. *The Lancet*'s editorial processes have only been applied to the original in English, which should serve as reference for this manuscript.

Terjemahan dalam Bahasa Indonesia ini diserahkan oleh penulis dan diproduksi kembali apa adanya tanpa melalui proses peer review. Proses editorial Lancet hanya diterapkan pada versi original dalam Bahasa Inggris, yang harus dijadikan rujukan untuk naskah ini.

Supplement to: RECOVERY Collaborative Group. Empagliflozin in patients admitted to hospital with COVID-19 (RECOVERY): a randomised, controlled, open-label, platform trial. *Lancet Diabetes Endocrinol* 2023; published online Oct 18. https://doi.org/10.1016/S2213-8587(23)00253-X.

Empagliflozin pada Pasien COVID-19 yang Dirawat Inap di Rumah Sakit (RECOVERY):

Uji Klinik Teracak, Terkontrol, Label Terbuka, Uji Klinik Berbasis Platform

Grup Kolaborasi RECOVERY*

RINGKASAN

Latar belakang : Empagliflozin telah diusulkan sebagai salah satu terapi COVID-19 berdasarkan efek anti inflamasi, efek metabolik dan hemodinamiknya. Uji Klinik RECOVERY bertujuan untuk menilai keamanan dan efikasi empagliflozin pada pasien COVID-19 yang dirawat di rumah sakit.

Metode : Dalam Uji Klinik Teracak, Terkontrol, Label Terbuka RECOVERY, beberapa obat uji dibandingkan dengan perawatan standar yang diberikan pada pasien COVID-19 yang dirawat inap di rumah sakit. Dalam analisis ini, kami mengkaji pasien dewasa yang memenuhi syarat dan setuju untuk berpartisipasi dalam uji klinik ini secara acak dialokasikan dengan rasio 1: 1 ke dalam kelompok perbandingan perawatan standar saja atau perawatan standar ditambah empagliflozin oral 10 mg sekali sehari selama 28 hari atau sampai pasien dipulangkan dari rumah sakit (mana yang terjadi lebih dulu), menggunakan pengacakan sederhana berbasis web (tidak bertingkat) dengan penyembunyian alokasi. Hasil primer yang diobservasi dalam uji klinik ini adalah kematian dalam 28 hari; sementara hasil sekundernya adalah durasi rawat inap dan risiko penggunaan ventilasi mekanis invasif atau risiko kematian (di antara peserta yang tidak menggunakan ventilasi mekanis invasif pada awal rawat inap). Pada 3 Maret 2023, *Data Monitoring Committee* independen merekomendasikan agar para peneliti melakukan tinjauan data uji klinik dan rekrutmen dihentikan pada 7 Maret 2023. Uji klinik RECOVERY yang sedang berlangsung, telah terdaftar di ISRCTN (50189673) dan *ClinicalTrials.gov*.(NCT04381936).

Hasil : Sejak 28 Juli 2021 hingga 6 Maret 2023, 4271 pasien secara acak dialokasikan untuk menerima obat empagliflozin (2113 pasien) atau perawatan standar saja (2158 pasien). Secara keseluruhan, 289 (14%) dari 2113 pasien yang dialokasikan untuk kelompok empagliflozin dan 307 (14%) dari 2158 pasien yang dialokasikan untuk kelompok perawatan

standar dilaporkan meninggal dalam kurun waktu 28 hari (*rate ratio* 0·96 [95% CI 0·82–1·13]; p=0·64). Tidak ada bukti perbedaan yang signifikan dalam durasi rawat inap (median 8 hari untuk dua kelompok tersebut) atau proporsi pasien yang keluar dari rumah sakit dalam keadaan hidup dalam waktu 28 hari (1678 [79%] pada kelompok empagliflozin vs 1677 [78%] pada kelompok perawatan standar saja; *rate ratio* 1·03 [95% CI 0·96–1·10]; p=0·44). Di antara mereka yang tidak menggunakan ventilasi mekanis invasif pada awal dirawat inap, tidak ada bukti perbedaan yang signifikan dalam proporsi yang memenuhi hasil akhir gabungan antara risiko penggunaan ventilasi mekanis invasif atau risiko kematian (338 [16%] of 2084 vs 371 [17%] of 2143; *risk ratio* 0·95 [95% CI 0·84–1·08]; p=0·44). Dua efek samping serius yang diyakini berhubungan dengan empagliflozin telah dilaporkan: keduanya merupakan efek samping berupa ketosis tanpa asidosis.

Interpretasi: Pada pasien COVID-19 dewasa yang dirawat inap di rumah sakit, penggunaan empagliflozin tidak berhubungan dengan penurunan angka kematian 28 hari, durasi rawat inap di rumah sakit, atau risiko berkembang menjadi membutuhkan ventilasi mekanis invasif maupun kematian sehingga tidak diindikasikan untuk perawatan pasien COVID-19 kecuali ada indikasi yang ditetapkan misalnya pada kondisi yang berbeda seperti diabetes.

Pendanaan : UK *Research and Innovation (Medical Research Council) and National Institute of Health Research* (MC_PC_19056), dan *Wellcome Trust* (222406/Z/20/Z).
