# Supplementary material for: Empagliflozin in patients admitted to hospital with COVID-19 (RECOVERY): a randomised, controlled, open-label, platform trial
Source: Lancet Diabetes Endocrinol. 2023 Dec;11(12):905–14. doi: 10.1016/S2213-8587(23)00253-X (PMC10957483; doi:10.1016/S2213-8587(23)00253-X)
Supplement: Vietnamese translation of the abstract [file mmc4.pdf]

THE LANCET
Diabetes & Endocrinology

Supplementary appendix 4

This translation in Vietnamese was submitted by the authors and we reproduce it as supplied. It has not been peer reviewed. *The Lancet*'s editorial processes have only been applied to the original in English, which should serve as reference for this manuscript.

Bản dịch sang tiếng Việt này do các tác giả gửi và chúng tôi sao chép lại như đã cung cấp. Bản dịch này không qua phản biện. Quy trình biên tập của The Lancet chỉ áp dụng cho bản gốc bằng tiếng Anh, và độc giả chỉ nên dùng bản gốc tiếng Anh làm tài liệu tham khảo.

Supplement to: RECOVERY Collaborative Group. Empagliflozin in patients admitted to hospital with COVID-19 (RECOVERY): a randomised, controlled, open-label, platform trial. *Lancet Diabetes Endocrinol* 2023; published online Oct 18. https://doi.org/10.1016/S2213-8587(23)00253-X.

Empagliflozin trên các bệnh nhân nhập viện vì COVID-19 (RECOVERY): một thử nghiệm nền tảng, ngẫu nhiên có đối chứng, nhãn mở

Nhóm cộng sự RECOVERY*

Tóm tắt

Tổng quan: Empagliflozin đã được đề xuất để điều trị COVID-19 dựa trên tác dụng chống viêm, tác dụng trên chuyển hóa và huyết động của thuốc. Thử nghiệm RECOVERY nhằm mục đích đánh giá tính an toàn và hiệu quả của Empagliflozin ở những bệnh nhân COVID-19 phải nhập viện.

Phương pháp: Trong thử nghiệm RECOVERY ngẫu nhiên có đối chứng, nhãn mở, một vài phương pháp điều trị tiềm năng đã được so sánh với chăm sóc thường quy ở các bệnh nhân nhập viện vì COVID-19. Trong lần phân tích này, chúng tôi đánh giá các bệnh nhân người lớn đáp ứng tiêu chí thu nhận và đồng ý tham gia nghiên cứu đã được phân ngẫu nhiên theo tỷ lệ 1:1 vào một trong hai nhánh: nhánh chỉ điều trị thông thường hoặc nhánh điều trị thông thường cộng thêm Empagliflozin 10mg đường uống một lần mỗi ngày cho đến khi xuất viện hoặc đến hết 28 ngày (tuỳ theo điều nào đến trước). Thử nghiệm sử dụng phương pháp phân ngẫu nhiên đơn giản (không phân tầng) tiến hành qua trang web, kết quả phân bổ không được biết trước. Kết cục chính là tỷ lệ tử vong trong vòng 28 ngày; các kết cục phụ là thời gian nằm viện và thông khí cơ học xâm lấn (trong số những người tham gia nghiên cứu không cần hỗ trợ thông khí xâm lấn ngay từ đầu) hoặc tử vong. Vào ngày 3 tháng 3, Ủy ban giám sát dữ liệu độc lập đã khuyến nghị các nghiên cứu viên xem xét dữ liệu đã có, sau đó việc thu tuyển bệnh nhân cho phương pháp điều trị này đã được dừng lại từ ngày 7 tháng 3. Thử nghiệm RECOVERY đang diễn ra được đăng ký với ISRCTN (50189673) và clinicaltrials.gov (NCT04381936).

Kết quả: Trong khoảng thời gian từ ngày 28 tháng 7 năm 2021 đến ngày 6 tháng 3 năm 2023, 4271 bệnh nhân đã được phân ngẫu nhiên vào nhánh điều trị bổ sung empagliflozin (2113 bệnh nhân) hoặc chăm sóc thường quy đơn thuần (2158 bệnh nhân). Dữ liệu về các kết cục chính và phụ đã được biết đến ở hơn 99% bệnh nhân được phân ngẫu nhiên.
Nhìn chung, 289 (14%) trong 2113 bệnh nhân có điều trị bổ sung empagliflozin và 307 (14%) trong 2158 bệnh nhân điều trị thông thường đã tử vong trong vòng 28 ngày (tỉ số tỉ suất mới mắc 0,96; [khoảng tin cậy 95% 0,82-1,13]; p=0,64). Không có bằng chứng về sự khác biệt đáng kể về thời gian nằm viện (trung vị 8 ngày cho cả hai nhóm) hoặc tỷ lệ bệnh nhân xuất viện còn sống trong vòng 28 ngày (1678 [79%] ở nhóm có điều trị bổ sung Empagliflozin so với 1677 [78%] ở nhóm chăm sóc thường quy đơn thuần; tỉ số tỉ suất mới mắc 1,03 [khoảng tin cậy 95% 0,96-1,10]; p=0,44). Trong số những người không cần thở máy xâm lấn ngay từ đầu, không có bằng chứng về sự khác biệt đáng kể về tỷ lệ tiến triển thành thở máy xâm lấn sau đó hoặc tử vong (338 [16%] trong 2084 so với 371 [17%] trong 2143; tỷ số nguy cơ 0,95 [khoảng tin cậy 95% 0,84-1,08]; p=0,44). Hai biến cố bất lợi nghiêm trọng được cho rằng có liên quan đến

empagliflozin đã được báo cáo: cả hai trường hợp chỉ nhiễm ceton mà không kèm theo nhiễm toan.

Biện giải: Đối với bệnh nhân người lớn phải nhập viện vì COVID-19, empagliflozin không làm giảm tỷ lệ tử vong trong vòng 28 ngày, thời gian nằm viện hoặc nguy cơ tiến triển đến thở máy xâm lấn hoặc tử vong, vì vậy thuốc không được chỉ định điều trị COVID-19 cho các bệnh nhân tương tự, trừ khi có một chỉ định phù hợp do một tình trạng khác như bệnh tiểu đường.

Tài trợ: Quỹ Nghiên cứu và Đổi mới của Vương quốc Anh (Hội đồng Nghiên cứu Y khoa) và Viện Nghiên cứu Sức khỏe Quốc gia (MC_PC_19056), và Wellcome Trust (222406/Z/20/Z).
